# Development and contextual adaptation of the first national clinical practice guideline for autism spectrum disorder in children and adolescents in Tunisia

**DOI:** 10.3389/fpsyt.2026.1926851

**Published:** 2026-10-20

**Authors:** Randaline Ayoub, Hela Ben Abid, Soumaya Bourgou, Imen Hadj Kacem, Soumeyya Halayem, Khaoula Khemekhem, Fatma Charfi, Asma Bouden

**Affiliations:** 1 Department of Child and Adolescent Psychiatry, Fattouma Bourguiba University Hospital, University of Monastir, Faculty of Medicine, Monastir, Tunisia; 2 Research Laboratory LR05ES10 “Vulnerability to Psychosis”, Monastir, Tunisia; 3 Department of Child and Adolescent Psychiatry, Mongi Slim University Hospital, University of Tunis El Manar, Faculty of Medicine, Tunis, Tunisia; 4 Research Laboratory ‘Mother-Baby Health’ LR22SP01, Tunis, Tunisia; 5 Department of Child and Adolescent Psychiatry, Hedi Chaker University Hospital, University of Sfax, Faculty of Medicine, Sfax, Tunisia; 6 Department of Child and Adolescent Psychiatry, Razi University Hospital, University of Tunis El Manar, Faculty of Medicine, Tunis, Tunisia

**Keywords:** adaptation, autism spectrum disorder, child and adolescent, practice guideline, Tunisia

## Abstract

**Introduction:**

Autism Spectrum Disorder (ASD) is a neurodevelopmental condition requiring long-term, coordinated, and multidisciplinary care. Although ASD services in Tunisia have improved, coordination across health, education, and social sectors remains limited, affecting equitable access and continuity of care. This study aimed to develop a national, evidence-based clinical practice guideline for the screening, diagnosis, and management of children and adolescents with ASD adapted to the Tunisian healthcare context.

**Assessment of guideline development and practice implications:**

The guideline was developed by the Tunisian Society of Child and Adolescent Psychiatry and accredited by the National Authority for Evaluation and Accreditation in Health. Using the ADAPTE framework, a multidisciplinary panel adapted international ASD guidelines to the Tunisian context. After systematic searches and AGREE II appraisal, the 2023 Singapore ASD guideline was selected as the main source document. Recommendations were adopted, adapted, or newly developed according to contextual relevance and applicability. An additional literature search was performed to incorporate high-level evidence published between June 2023 and January 2025.

**Actionable recommendations:**

The final guideline includes 96 evidence-based recommendations and 87 good practice points organized into ten chapters, covering the screening, diagnosis, intervention, and follow-up of autism spectrum disorder in children and adolescents across all levels of care in Tunisia. This article summarizes the key practice-oriented recommendations across these chapters.

**Conclusion:**

This first Tunisian ASD guideline aims to harmonize practices, improve the quality of care, and strengthen integrated care pathways.

## 1 Introduction

Autism Spectrum Disorder (ASD) is a heterogeneous neurodevelopmental condition characterized by differences in communication and social interaction, as well as restricted and repetitive behaviors (1). The World Health Organization (WHO) has estimated the prevalence of autism in children at approximately 1 in 100 (2), although this figure varies considerably across populations, reflecting differences in diagnostic practices, case identification, and access to assessment and services (3). In Tunisia, prevalence aligns with current global estimates. A descriptive multicenter cross-sectional study conducted in 2022 included a sample of 918 children aged 18 to 36 months and reported an ASD prevalence of 1.01% (95% CI [0.35–1.65]) (4).

The impact of ASD extends beyond the affected individual, profoundly influencing families and the broader community. Individuals with ASD often require long-term, multidisciplinary support, while caregivers face considerable ongoing demands. Early diagnosis and timely, evidence-based interventions can improve developmental outcomes, social participation, and quality of life for both children and their families (1).

In Tunisia, the management of children with ASD has evolved considerably over the years, particularly due to improvements in diagnostic tools and increased awareness among healthcare professionals and the general public. These advances have enabled better identification of affected children and earlier referral to appropriate and targeted interventions. In Tunisia, child and adolescent mental health services face important organizational challenges, including the unequal geographical distribution of specialized professionals and limited coordination between the health, education and social sectors (5). These challenges highlight the need for harmonized national clinical practice guidelines to support more equitable and coordinated ASD care.

Despite these efforts, Tunisia currently lacks standardized national guidelines for ASD. In this context, the development of a clinical practice guideline (CPG) adapted to the Tunisian context, compiling best practices, was considered necessary.

This guideline represents the first Tunisian CPG on ASD. It was developed under the auspices of the Tunisian Society of Child and Adolescent Psychiatry (STPEA). It received the official National Authority for Evaluation and Accreditation in Healthcare (INEAS) label following independent methodological appraisal and clinical review.

The objective of this guideline was to provide a national, evidence-based framework for the screening, diagnosis, and management of children and adolescents with ASD, adapted to the Tunisian healthcare context.

This guideline addresses children and adolescents with ASD, regardless of severity, as well as those at increased risk for ASD. While certain sections may consider transition to adulthood, the primary focus is on individuals under 18 years, consistent with Tunisia’s legal definition of childhood. Particular attention is given to subgroups with specific needs, including girls with ASD, children from diverse cultural backgrounds, and those from socioeconomically disadvantaged families. The guideline is intended for healthcare and education professionals involved in the care of children with ASD, including pediatricians, child and adolescent psychiatrists, neurologists, general practitioners, neonatologists, otolaryngologists, geneticists, psychologists, nurses, speech and language therapists, occupational therapists, psychomotor therapists, special education teachers, and pediatric nurses. It is also relevant to social workers, caregivers, policymakers, patient representatives, and non-governmental organizations, including professional societies and associations.

## 2 Assessment of guideline development and practice implications

For the development of this guideline, we used the ADAPTE methodological tool, which enables the adaptation of existing recommendations to the Tunisian context while respecting the fundamental principles of evidence-based medicine (6). The development and updating of high-quality guidelines require considerable investment in time and resources. Adapting existing recommendations allows alignment with international practices and helps optimize efficiency.

The ADAPTE tool is structured into three main phases: set-up, adaptation, and finalization.

### 2.1 Protocol development and approval

The guideline development and adaptation protocol was approved by the National Authority for Evaluation and Accreditation in Healthcare (INEAS), Tunisia, prior to the initiation of the development process. The protocol outlined the objectives, the PIPOH question, the methodological framework based on the ADAPTE process, the literature search strategy, eligibility criteria, appraisal methods (AGREE II), and the approach to recommendation grading. The development process was conducted in accordance with the approved protocol. The protocol is available from the corresponding author upon reasonable request.

### 2.2 Set-up

The set-up phase aimed first to define the PIPOH question to be addressed before initiating the CPG development process.

The STPEA requested the support of the INEAS in developing a CPG on the screening, diagnosis, and management of autism spectrum disorder (ASD) in Tunisia. In this context, a group of child psychiatrists participated in a training session organized by INEAS (Directorate of Quality of Care and Patient Safety: QSSP), during which the PIPOH question was developed according to the scope of the guideline. The PIPOH framework was used to define the target population, interventions, professionals involved, expected outcomes, and healthcare setting of the guideline (**Table 1**). A first meeting with the working group was held in March 2024 to present the objectives of the project, the methodology to be followed, and validate the PIPOH question.

**Table 1:** The PIPOH model.

PIPOH component	Description
P: Population	Children and adolescents with ASD
I: Intervention	Screening, diagnosis, treatment, and follow-up
P: Professionals	Child psychiatrist, psychiatrist, pediatrician, pediatric neurologist, neonatologist, geneticist, ENT specialist, general practitioner, biochemist, speech therapist, psychologist, psychomotor therapist, and occupational therapist
O: Outcomes	Early diagnosis and intervention, access to care, management, and follow-up
H: Healthcare setting	All levels of care (public and private sectors)

#### 2.2.1 Formation of the working group

All scientific societies involved in the screening, diagnosis, management, and follow-up of ASD were contacted to form the expert panel representing the working group.

Clinical experts were appointed to serve as president, vice-president, general secretaries, and section heads. The working group consisted of child psychiatrists, psychiatrists, pediatricians, pediatric neurologists, neonatologists, geneticists, otorhinolaryngologists (ENT specialists), general practitioners, biochemists, speech therapists, psychologists, psychomotor therapists, occupational therapists, and pediatric nurses.

The methodological team consisted of two child psychiatrists who had received specific training provided by the INEAS team.

#### 2.2.2 Literature search

A search for CPGs was conducted by the methodological team in March 2024 through the Guidelines International Network (GIN) and the websites of governmental agencies: the National Institute for Health and Care Excellence (NICE), the Scottish Intercollegiate Guidelines Network (SIGN), the International Association for Child and Adolescent Psychiatry and Allied Professions (IACAPAP), and the European Society for Child and Adolescent Psychiatry (ESCAP), as well as in the databases “PubMed,” “Trip Database,” and “Embase”. Search strategies were developed using the following keywords: “autism,” “autism spectrum disorder,” “autistic disorder,” “infantile autism,” “childhood autism,” “practice guidelines,” “clinical practice guidelines,” and “evidence-based medicine.” The search identified 268 records from bibliographic databases and 27 records from guideline repositories/websites, yielding 295 records in total before deduplication. Records identified through bibliographic databases and guideline repositories/websites were pooled prior to deduplication and screening, as several guidelines retrieved from guideline repositories/websites were also independently identified through the database searches. Duplicate records were therefore checked jointly across all sources. Detailed search strategies, search dates, publication periods, and the number of records retrieved from each source are provided in **Supplementary Table S1**.

Guidelines were eligible if they addressed the screening, diagnosis, and management of autism spectrum disorder in children and adolescents, were available in full text, published between 2015 and 2024, and written in English or French. Documents focusing exclusively on adults, consensus statements without a systematic evidence review, adapted guidelines derived from other existing guidelines, outdated versions superseded by more recent guidelines, and publications that did not provide explicit clinical recommendations were excluded.

The guideline search, screening, and quality appraisal were independently performed by two trained methodologists. Any disagreements regarding eligibility, quality assessment, or guideline selection were resolved through discussion and consensus. Eleven guidelines were assessed in full text; five met the predefined eligibility criteria and were retained for AGREE II appraisal, while six were excluded because they did not fully satisfy one or more predefined PIPOH criteria (**Figure 1**). The excluded guidelines and the specific PIPOH criteria not met are presented in **Supplementary Table S2**. The guideline selection process was reported in accordance with the PRISMA 2020 statement (7).

**Figure 1:**
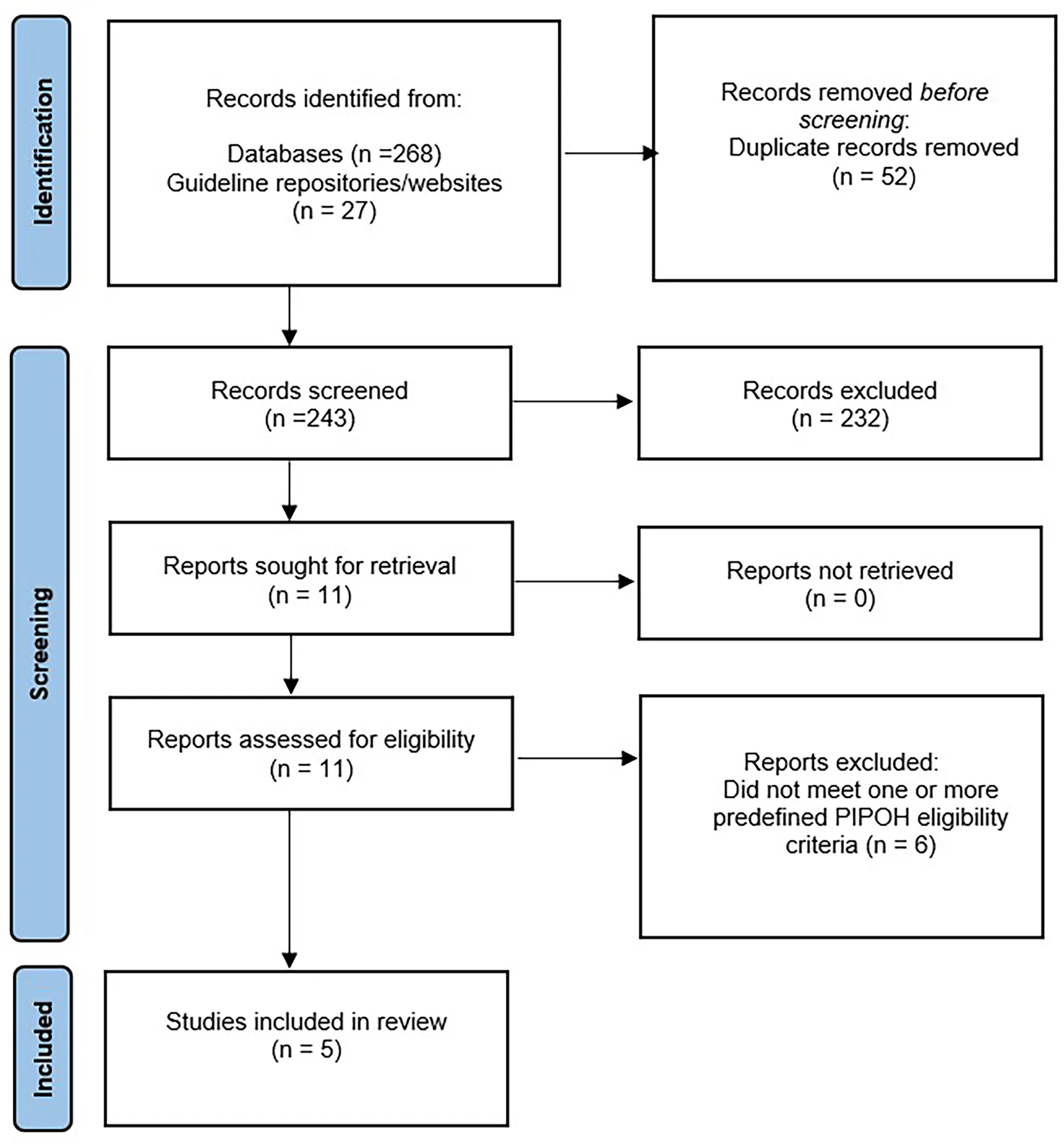
PRISMA flow diagram *Note.* Records from bibliographic databases and guideline repositories/websites were pooled before deduplication because of overlap between these sources.

A supplementary search was conducted through January 2025 to identify relevant CPGs published after the initial search. A further supplementary search was conducted in September 2026 during the current manuscript revision to identify any additional relevant CPGs. No additional eligible CPG was identified.

#### 2.2.3 Declaration of conflicts of interest

Members of the expert panel invited to participate in the working and review groups submitted declarations of conflicts of interest.

### 2.3 Adaptation

The adaptation process followed the ADAPTE framework and included methodological appraisal of the pre-selected guidelines, selection of a primary source guideline, assessment of the consistency, acceptability, and applicability of recommendations.

#### 2.3.1 Evaluation of guidelines on autism spectrum disorder

The quality of the five pre-selected guidelines (the NICE guideline, the SIGN guideline, the Australian guideline, the Singapore guideline, and the ESCAP guideline) was assessed using the “Appraisal of Guidelines for Research & Evaluation II” (AGREE II) tool. Two trained methodologists independently appraised the methodological quality of the five pre-selected guidelines using the official AGREE II Online Guideline Appraisal Tool through the AGREE Enterprise platform (AGREE Trust). Each methodologist entered their ratings directly into the online tool, which automatically calculated the standardized percentage scores for the six AGREE II domains according to the standard AGREE II scoring method. The Overall Assessment reported in **Table 2** was also obtained directly from the online tool and represents the appraisers’ overall assessment of each guideline; it was not calculated as an average of the six domain scores. Given the limited number of appraisers (n = 2) and guidelines (n = 5), a formal statistical measure of inter-rater agreement was not computed; instead, discrepancies between the two independent scores were identified item-by-item and resolved through discussion until full consensus was reached. The AGREE II domain scores and Overall Assessment for all five pre-selected guidelines are presented in **Table 2**.

**Table 2:** AGREE II appraisal scores of the pre-selected guidelines.

Guideline	Scope & purpose	Stakeholder involvement	Rigour of development	Clarity of presentation	Applicability	Editorial independence	Overall assessment
NICE	100%	97%	97%	94%	75%	88%	92%
Singapore	94%	100%	93%	94%	81%	88%	83%
Australian	94%	94%	88%	83%	77%	83%	83%
SIGN	100%	83%	84%	92%	88%	88%	83%
ESCAP	72%	14%	31%	42%	17%	71%	33%

Domain scores and Overall Assessment were generated by the official AGREE II Online Guideline Appraisal Tool.

Four of the five pre-selected guidelines achieved more than 66% in AGREE II Domain 3 (Rigour of Development), whereas the ESCAP guideline scored 31% in this domain and was therefore not retained as the primary source. Although both the NICE and Singapore guidelines demonstrated high methodological quality, the working group selected the Singapore guideline as the primary source document. This decision was not based on the Overall Assessment alone, but on the combined consideration of methodological rigour, recency, comprehensiveness, and alignment with the predefined PIPOH framework (8). In particular, the Singapore guideline, updated in June 2023, provided the most recent guideline among the eligible guidelines and showed strong alignment with the predefined PIPOH criteria. It was also structurally more suitable as a primary source: it is a single, integrated guideline, whereas the relevant NICE material for children and adolescents with ASD is distributed across two separate guidelines of differing vintages (CG128 (2011) and CG170 (2013)). This selection was consistent with the ADAPTE approach, which supports the use of recent, methodologically robust source guidelines as a basis for contextual adaptation. Although the ADAPTE framework allows the integration of recommendations from multiple source guidelines, a single primary source guideline was selected to ensure methodological coherence and consistency throughout the adaptation process.

An official request for authorization to proceed with the adaptation of this guideline was submitted by the STPEA to the College of Paediatrics and Child Health, Singapore, and approval was granted.

#### 2.3.2 Analysis of consistency, acceptability, and applicability of the recommendations

The methodological team assessed each recommendation from the source guideline using ADAPTE Tools 14 and 15. First, the consistency between the scientific evidence and the recommendation was evaluated. Subsequently, the acceptability and applicability of each recommendation within the Tunisian healthcare context were assessed. This contextual assessment considered the availability of specialists, regional disparities in service provision, the organization of the Tunisian healthcare and education systems, the availability of validated screening and diagnostic tools, locally approved pharmacological treatments, existing intervention programmes, and the feasibility of implementation within available resources. Based on this structured assessment, recommendations were classified as adopted without modification, adapted to the Tunisian context, or developed *de novo* when no suitable source recommendation was available or when contextual factors required formulation of a new recommendation. Final decisions were reached by multidisciplinary consensus during dedicated working-group meetings.

For recommendations requiring adaptation or *de novo* development, an additional literature search was performed to assess contextual relevance and incorporate high-level evidence published between June 2023 (publication of the Singapore guideline) and January 2025, thereby ensuring that the adapted guideline reflected the most recent available evidence.

#### 2.3.3 Drafting of the guideline

The drafting of the adapted guideline (French version) was entrusted to four working groups, each composed of professionals from the specialties mentioned above. The principle of parity between the group of child psychiatrists (50%) and the multidisciplinary group was respected. A work schedule and timeline of meetings were established for each stage of the process.

The rationale supporting each adaptation decision was systematically documented during the adaptation process using standardized evidence tables summarizing the source recommendation, contextual considerations, supporting evidence, and the justification for adoption, adaptation, or *de novo* development.

The grading framework used to classify the strength of the recommendations is presented in **Table 3**. Recommendation codes consist of the recommendation category (R = Strong Recommendation; RC = Conditional Recommendation; GPP = Good Practice Point), followed by the chapter number and the sequential recommendation number within that chapter (e.g., R 1.10; RC 3.2; GPP 1.7). The R/GPP distinction was derived from the source Singapore guideline (8). The working group additionally introduced the explicit “RC” (Conditional Recommendation) code to formalize a distinction already reflected in Singapore’s modal verb phrasing (e.g., “should/must” versus “may”). For recommendations adopted unchanged, the level of evidence and recommendation strength were initially derived from the Singapore guideline (8). Using the contextual assessment performed with ADAPTE Tools 14 and 15, recommendation strength was subsequently reassessed in light of additional evidence and the Tunisian context, including clinical relevance, feasibility, available resources, and applicability. Therefore, the strength of a recommendation could differ from the level of evidence following contextual reassessment. The original grading was retained only when considered appropriate after this reassessment. Recommendations developed *de novo* were formulated as GPP, with their content and wording determined through multidisciplinary expert consensus, considering the available evidence, clinical relevance, feasibility, available resources, and applicability to the Tunisian healthcare system.

**Table 3:** Grading of recommendations and evidence levels.

Category	Evidence Level	Recommendation Statement
R – Strong Recommendation	High-quality evidence generally supporting a strong recommendation, with consideration of clinical relevance, feasibility, available resources, and applicability to the Tunisian context	“it is/is not recommended to…” “it must/must not be…” “it should/should not be…”
RC – Conditional Recommendation	Moderate- or low-quality evidence, or evidence for which a conditional recommendation is more appropriate considering clinical relevance, feasibility, available resources, and applicability to the Tunisian context	“It may be …”
GPP – Good Practice Point	Expert agreement/Professional consensus	“Based on expert consensus, it is recommended to…”

*The R/GPP distinction was derived from the 2023 Singapore Clinical Practice Guideline on Autism Spectrum Disorder. The RC category was introduced by the guideline working group to formalize conditional recommendations.

The contextual adaptation resulted in a combination of adopted, adapted, and *de novo* statements across the 10 chapters of the Tunisian guideline. In the screening and diagnostic assessment sections, Singapore-specific tools or references were localized to the Tunisian context, as illustrated by GPP1.4. In the etiological investigations section, GPP2.10 was developed *de novo* to address awareness of the potential negative effects of screen exposure in children with autism. In pharmacological treatment, recommendations were contextualized according to medication availability and national regulatory status; for example, RC 4.2 was adapted to retain risperidone while excluding aripiprazole for the indication concerned. In education, GPP5.1 and GPP5.2 were adapted to the Tunisian healthcare and educational context, while GPP5.8 was developed *de novo* to reflect the Tunisian framework for inclusive education.

To summarize the extent of contextual adaptation, 157 statements (85.8%) were adopted, 20 (10.9%) were adapted, and 6 (3.3%) were developed *de novo*. Representative examples illustrating the contextualization process are provided in **Supplementary Table S3**.

Draft recommendations were discussed during structured multidisciplinary working-group meetings. When disagreements arose, recommendations were revised following discussion until consensus was achieved. The final wording and adaptation decisions were validated by all members of the guideline development group before external review.

### 2.4 Finalization

The finalization phase included external review, followed by methodological and scientific review, and planning for implementation, monitoring, and future updating.

#### 2.4.1 External review

An external multidisciplinary reading group of 17 members was established to review the draft guideline. The group included specialists in child and adolescent psychiatry, psychiatry, paediatric neurology, neurology, neonatology, genetics, pharmacy, primary care, rehabilitation, school health, psychology, and education. It also included an autistic service user and the service user’s parents/caregivers, as well as a representative of the Ministry of Education. Reviewers provided written comments and feedback on the draft guideline and recommendations; no formal appraisal tool was used for this external reading process. Their feedback was considered with regard to the acceptability, applicability, and contextual relevance of the guideline.

The Ministry of Education representative provided additional information on the Tunisian educational framework, including school inclusion measures, individualized educational plans, examination accommodations, and implementation barriers related to school support. For example, following this feedback, GPP 5.3 was revised to explicitly incorporate the Individualized Education Plan when considering the child’s developmental needs, preferences, strengths, family context, and available educational measures.

Following the external review and subsequent revisions, the guideline underwent methodological and scientific review by a six-member expert committee of the INEAS, comprising methodological and clinical experts. The committee used the AGREE II instrument to appraise the revised guideline and provided additional comments and recommendations. The guideline was further revised as appropriate based on this review before receiving the official INEAS label.

## 3 Actionable recommendations

This section presents a summary of the key recommendations from the Tunisian CPG on Autism Spectrum Disorder for children and adolescents. These recommendations were selected to provide a concise overview of the principal clinical messages across the ten chapters. For clarity and readability, they are grouped by clinical theme rather than presented in their original numerical order. The complete list of recommendations and Good Practice Points is available in **Supplementary File S4**. The full guideline is available on the official STPEA and INEAS websites.

### 3.1 Screening and diagnosis

Early identification of autism is essential to allow timely referral and early intervention, which may improve long-term developmental outcomes (GPP 1.1) (9, 10).

Monitoring for early signs of autism should therefore be integrated into a national developmental surveillance program (GPP 1.2).

Children presenting with specific clinical warning signs should be promptly referred for a comprehensive developmental evaluation (GPP 1.7) (11). These clinical features are summarized in **Table 4**.

**Table 4:** Clinical features requiring prompt referral.

Clinical feature (Red flag)
• Any regression or loss of language or social skills.
• Absence of babbling, use of social gestures (e.g., waving, saying “bye-bye,” pointing), shared enjoyment (spontaneously showing objects, following pointing or gaze), or response to name by around 12 months of age.
• Absence of single communicative words, lack of response to simple instructions, or absence of pretend play by 18 months.
• Reduced eye contact or social responsiveness.
• Presence of unusual, repetitive, rigid, or obsessive behaviours.
• Sensory abnormalities, including hypo- or hypersensitivity to sensory stimuli, at any age.

Developed by the guideline working group.

Healthcare professionals should also be aware of factors associated with an increased likelihood of autism and may consider targeted screening in children presenting developmental concerns or these risk factors (GPP 1.9) (12, 13). These factors are summarized in **Table 5**.

**Table 5:** Factors associated with an increased likelihood of autism.

Risk factor
• A history of autism in a sibling.
• Prematurity <35 weeks of gestation or birth weight <2500 g.
• Neonatal hypoxic-ischemic encephalopathy.
• A Known genetic syndrome associated with autism.
• *In-utero* exposure to maternal antiepileptic medication.
• Advanced parental age at birth (>40 years).
• Parental history of mental health disorders**.

*Adapted from the 2023 Singapore Clinical Practice Guideline on Autism Spectrum Disorder and contextualized for the Tunisian guideline.

**Parental history of mental health disorders is intended solely as a prompt for heightened developmental vigilance, to be applied in a non-stigmatizing manner and not as a criterion for diagnosis or access to services.

The systematic use of autism-specific screening tools in the general pediatric population without identified risk factors is not recommended (R 1.10) (14). However, when developmental delay or other developmental concerns are suspected, autism-specific screening tools may be used to complement the clinical judgment of healthcare professionals. These tools should not replace clinical assessment and cannot confirm or exclude a diagnosis of autism on their own (R 1.11) (15).

Screening instruments should be used only within the age ranges for which they have been validated. Healthcare professionals should be aware that the diagnostic accuracy of screening tools is generally higher in children aged 21 months and older than in those aged 12–20 months (R 1.13).

Professionals involved in the diagnosis of autism in children and adolescents should rely on internationally recognized diagnostic classification systems and use the most recent versions of the Diagnostic and Statistical Manual of Mental Disorders or the International Classification of Diseases. The classification system used should be clearly specified (GPP 1.15).

Assessment and diagnosis should not rely solely on autism-specific diagnostic instruments. A comprehensive evaluation should establish a holistic developmental profile of the child, including developmental, medical, and social history; neurological and physical examination; consideration of differential diagnoses and comorbid conditions; and assessment of cognitive, sensory, academic, behavioral, and adaptive functioning. In addition, the child’s strengths, abilities, and needs should be identified to guide intervention planning. Autism-specific diagnostic instruments may complement clinical observation and information gathering during the assessment process (R 1.20).

### 3.2 Etiology and diagnostic investigations

Healthcare professionals should be aware of the strong genetic heritability of autism and monitor for autistic traits in children with first-degree relatives (siblings or parents) with autism spectrum disorder (GPP 2.1). Several genetic disorders are known to be associated with autism, and affected children should be monitored for relevant signs (GPP 2.2). The main genetic disorders frequently associated with autism are summarized in **Table 6** (16, 17).

**Table 6:** Main genetic disorders associated with ASD.

Genetic disorder
• Fragile X syndrome
• Angelman syndrome
• Tuberous sclerosis complex (Bourneville disease)
• Rett syndrome
• PTEN hamartoma tumor syndrome
• Trisomy 21

*Adapted from the 2023 Singapore Clinical Practice Guideline on Autism Spectrum Disorder and contextualized for the Tunisian guideline.

Current evidence does not support a causal association between autism and maternal use of paracetamol during pregnancy, epidural analgesia during labour, or childhood vaccinations. Pregnant women may use paracetamol when clinically indicated, epidural analgesia should not be avoided when necessary, and children should follow the recommended immunization schedule, including the MMR vaccine (GPP 2.3, 2.5, 2.6) (18, 19).

Healthcare professionals should discuss the indications and potential adverse effects of antiepileptic medications with women of childbearing age, as prenatal exposure to sodium valproate has been associated with an increased risk of autism (GPP 2.4) (20).

Routine screening for heavy metal levels (antimony, aluminum, arsenic, cadmium, lead, manganese, mercury, nickel, silver, thallium) is not recommended for children with autism, as no causal relationship has been established (GPP 2.7). However, testing for mercury toxicity may be considered in children with severe neurological or immunological symptoms (GPP 2.8), and screening for lead toxicity may be considered in children with suspected pica or other feeding disorders (GPP 2.9).

Healthcare professionals should also be aware of the potential negative effects of excessive screen exposure in children with autism (GPP 2.10).

In the absence of an obvious etiology, a minimal metabolic work-up may be considered based on clinical orientation. Basic metabolic investigations are summarized in **Table 7**.

**Table 7:** Basic metabolic investigations in the absence of identified etiology.

Investigation
• Ammonia level
• Uric acid
• Plasma phenylalanine measurement
• Amino acid chromatography
• Homocysteine measurement
• Organic acid chromatography
• Urinary guanidinoacetate measurement and creatine/creatinine ratio

Developed by the guideline working group.

### 3.3 Intervention

Children and adolescents with ASD may benefit from a range of interventions targeting communication, social interaction, emotional regulation, and adaptive functioning. Evidence syntheses of early interventions in young children with ASD indicate beneficial effects for some developmental and behavioral outcomes, although the magnitude and consistency of effects vary across intervention types, outcomes, and individual characteristics (21). Augmentative and Alternative Communication (AAC) can be used to support both receptive and expressive communication, and should be tailored to the individual’s needs, preferences, communication mode, and environment (R 3.1) (22). Communication-focused interventions, such as speech and language therapy or Pivotal Response Training, may enhance joint attention, social engagement, initiation, receptive and expressive language, and speech prosody (RC 3.2). Developmental interventions delivered according to a developmental sequence and emphasizing skill acquisition through interactions with caregivers may further improve communication and social skills (RC 3.3).

Behavioral interventions can also be considered (23, 24). Early Intensive Behavioral Intervention, delivered by qualified professionals with sufficient intensity and predefined goals for the child and family, may improve adaptive skills and cognitive abilities (RC 3.4). Naturalistic Developmental Behavioral Interventions, which combine behavioral and developmental approaches in natural environments using child-centered, motivation-based strategies during play and daily routines, may enhance social communication, language, cognitive, and play skills (RC 3.6) (25). Play-based interventions, including structured games, toys, and materials, can promote play, joint attention, language, and social engagement, especially in children under 12 years of age, whereas play therapy may address emotional and behavioral difficulties (RC 3.7).

Emotional and cognitive interventions include Emotional Regulation Therapy, which may use computer programs, videos, or games to teach emotion recognition and regulation through a social-pragmatic approach (RC 3.5) (26). Cognitive Behavioral Therapy may be applied for children and adolescents with adequate verbal and reasoning abilities to address anxiety, anger, or other emotional difficulties, with adaptations as needed and parental involvement to support generalization (RC 3.13) (27).

Sensory and environmental interventions can be considered to support functional participation. Sensory integration therapy incorporating elements described by Ayres may be recommended for children aged 3–12 years to improve social communication and participation (RC 3.8) (28, 29), while modifications of the sensory environment and sensory modulation strategies may help address specific sensory needs (RC 3.9). The use of weighted vests is not recommended because of insufficient evidence of benefit and concerns regarding potential risks (R 3.10).

Social skills interventions should be offered to improve social communication and interaction, and may also have positive effects on challenging behaviors, adaptive and cognitive abilities, and academic performance. These interventions should be individualized according to each child’s needs, preferences, and environment (R 3.11) (27, 30). Visual supports, including pictures, objects, written words, lists, schedules, or choice boards, should be used to enhance communication and routine understanding (R 3.12).

### 3.4 Pharmacological treatment

Pharmacological interventions may be considered for children and adolescents with ASD primarily to manage co-occurring conditions or specific challenging behaviors. Methylphenidate should be considered as a first-line treatment for attention-deficit/hyperactivity disorder in this population, always in combination with non-pharmacological interventions (R 4.1) (31). Risperidone may be used in the short-term to manage irritability or hyperactivity, but evidence is insufficient to support its long-term use beyond six months, and it may cause weight gain and sedation (RC 4.2) (32). Melatonin may be considered for sleep disorders when psychosocial interventions are ineffective, in combination with a behavioral intervention delivered by a qualified specialist (RC 4.9) (33).

Selective serotonin reuptake inhibitors, such as fluoxetine, sertraline, escitalopram, or paroxetine, should not be used to treat core symptoms of autism but may be prescribed for co-occurring psychiatric disorders, including anxiety, depression, or obsessive-compulsive disorder by a qualified specialist (R 4.3) (34). Similarly, tricyclic antidepressants may be considered only for psychiatric disorders and should not be used to manage challenging behaviors (R 4.4) (35). Antiepileptic drugs or mood stabilizers should not be routinely used for behavioral management, though they may be considered as second- or third-line options for psychiatric comorbidities under specialist supervision (R 4.5) (36). The combination of buspirone, celecoxib, or galantamine with risperidone for challenging behaviors is not recommended (R 4.6–4.8).

A wide range of pharmacological agents should not be used to treat the core symptoms of autism, including acamprosate, amantadine, arbaclofen, bumetanide, D-cycloserine, memantine, N-acetylcysteine, riluzole, intranasal oxytocin, balovaptan, IGF-1 analogues (e.g., trofinetide, mecasermin), mTOR inhibitors (everolimus, rapamycin), metformin, cholinesterase inhibitors, cannabinoids, suramin, and piracetam. Many of these agents also should not be used as adjuncts to risperidone for irritability due to insufficient evidence, and further clinical trials are awaited for several compounds (R 4.10–4.27).

### 3.5 Education

Educational recommendations for children with ASD in Tunisia emphasize school inclusion and individualized support, with educational accommodations based on the specific needs of each student rather than solely on diagnosis (GPP 5.8, 5.10) (37). Educational interventions should be tailored to the child’s developmental profile, strengths, and preferences, while also considering the family context and available services (GPP 5.3) (38).

Parents are encouraged to consult qualified professionals when considering educational interventions and school inclusion and should be supported in making informed decisions regarding their child’s long-term educational needs (GPP 5.1, 5.2, 5.4). Teachers should have appropriate knowledge and training to provide necessary educational accommodations, and educational decisions should involve collaboration between parents and professionals (GPP 5.9, 5.11). Parents should also receive early information about school inclusion measures, generally around the age of five years (GPP 5.6).

When children cannot be integrated into mainstream school settings, referral to specialized centers offering intensive and individualized interventions may be considered (GPP 5.13). School transitions should be carefully planned and supported to promote adaptation and reduce stress, with coordinated collaboration between families, schools, and professionals (GPP 5.5, 5.7, 5.14, 5.15, 5.18). School support assistants can play an important role in facilitating inclusion and supporting learning in the classroom (GPP 5.12). Finally, adolescents with ASD and their families should receive information about post-school pathways and employment support to promote successful psychosocial and vocational integration (GPP 5.19).

### 3.6 Complementary and alternative treatments

The evidence for complementary and alternative interventions varies across approaches, with some supported by clinical evidence while others lack sufficient evidence to support their routine use. Most complementary and alternative interventions are not recommended for treating the core symptoms of autism in children and adolescents. Diet-based approaches, including gluten-free or casein-free diets, ketogenic diets (except when indicated for drug-resistant epilepsy under dietitian supervision), and camel milk, have no proven benefit. Similarly, vitamin and mineral supplementation, such as vitamins B6, B12, folinic acid, zinc, magnesium, iron, and other minerals: amino acids, omega-3 fatty acids, probiotics, secretin, digestive enzymes, sulforaphane, and coenzyme Q10 are not supported by evidence for treating core autism symptoms (R 6.2, 6.4–6.17) (39). However, children and adolescents with autism spectrum disorder presenting symptoms suggestive of nutritional deficiencies should be appropriately assessed, treated, and monitored according to relevant clinical guidelines (GPP 6.7).

Pharmacological-like or experimental interventions, including antimicrobial therapy, microbiota transfer therapy, helminth therapy, mesalazine, immunoglobulin therapy, stem cell therapy, hyperbaric oxygen therapy, chelation therapy, neurofeedback, vagus nerve stimulation, and transcranial direct current stimulation, are also not recommended due to lack of benefit and potential harm (R 6.18–6.29) (40, 41).

Behavioral, sensory, and body-based therapies, including dance and movement therapy (R 6.31), art therapy (R 6.33), aromatherapy (R 6.36), acupuncture (R 6.37), massage therapy (R 6.38), chiropractic care, osteopathy, craniosacral therapy (R 6.40), animal-assisted therapy (R 6.42), and facilitated communication (R 6.45), should not be used as treatments for core autism symptoms. Evidence is insufficient, and some of these approaches may pose risks (42, 43).

A few interventions may be considered as complementary to support overall well-being without being treatments for core autism symptoms. Music therapy may improve global functioning, quality of life, and reduce autism severity, and may be offered as part of a comprehensive care plan (RC 6.30) (44, 45). Physical activity should be encouraged, tailored to the child’s age and developmental abilities, in line with WHO recommendations (R 6.41). Mindfulness may also be included for general well-being, although it is not a treatment for core autism symptoms (RC 6.44). Finally, selective visuomotor exercises may be beneficial for children with visual difficulties to improve social communication and reduce repetitive behaviors (RC 6.35).

### 3.7 Autism and associated conditions

Children and adolescents with ASD should receive regular, holistic follow-up due to increased risks of neuropsychological, adaptive, academic, and medical difficulties (GPP 7.1) (46). Adaptive functioning should be assessed using standardized tools, even in the absence of cognitive impairment, to guide support for daily life (R 7.2). Academic difficulties should prompt tailored assessments to inform interventions and learning accommodations (R 7.3).

Neurodevelopmental and motor comorbidities are common (47). Attention-deficit/hyperactivity disorder should be screened for and evaluated comprehensively when symptoms are present, particularly after age five (R 7.4). Motor difficulties may indicate Developmental Coordination Disorder and require standardized evaluation (R 7.5). Global developmental delay should be reassessed before school age to determine intellectual disability (R 7.7). Language, learning, and other developmental disorders should also be monitored to guide appropriate support (R 7.6).

Sensory, mental health, and behavioral issues are frequent. Sensory processing difficulties should be evaluated holistically (R 7.8). Anxiety, depression, and other psychiatric comorbidities should be assessed by trained professionals (R 7.9). Eating and feeding problems, including pica, rumination, obesity, and food neophobia, should be evaluated when clinically indicated (R 7.11). Sleep patterns should be monitored and managed, as poor sleep affects both the child and caregivers (R 7.13).

Medical comorbidities such as gastrointestinal disorders and hearing impairment are also more prevalent. Persistent gastrointestinal symptoms require further evaluation (R 7.16), and a comprehensive hearing assessment is recommended for all children with suspected ASD (R 7.20).

### 3.8 Follow-up and prognosis

Long-term outcomes in ASD are influenced by several factors. Poorer prognosis is associated with greater severity of autistic traits in childhood, co-occurring intellectual disability, and delayed language development. Because individuals with ASD may experience persistent difficulties affecting adaptive functioning, emotional well-being, and academic achievement, interventions should address these needs across the lifespan and consider the goals of the child or adolescent and their family (GPP 8.2) (48).

Supportive family environments and positive maternal and paternal parenting are associated with improved developmental outcomes. Interventions that promote positive parenting and provide parents with early information, training, and support should therefore be encouraged (GPP 8.1) (49).

Transition to adulthood is a critical period, often marked by difficulties with autonomy, high rates of psychiatric comorbidities, and challenges in social and vocational integration. Early, proactive, and person-centered transition planning, particularly before leaving school or specialized services, is essential to support psychosocial rehabilitation, vocational training, and supported employment (GPP 8.3).

Data on long-term outcomes for adults with ASD remain limited in Tunisia, highlighting the need for further research.

### 3.9 Support for caregivers and families

Professionals should adopt a collaborative, family-centred approach to help parents/caregivers achieve optimal therapeutic outcomes (GPP 9.3). They should also assess the emotional well-being and coping capacity of parents/caregivers to provide the support needed for holistic care (GPP 9.4). Parent/caregiver training and education should be integrated into intervention programs for children and adolescents with ASD whenever possible, given their positive effects on both the child or adolescent and the caregiver (R 9.6) (50).

### 3.10 Professional training

All professionals involved in the care of children and adolescents with ASD, whether frontline providers, specialists, or allied professionals, should have access to appropriate training on autism. This training should include information on neurodiversity and be tailored to the specific needs of the professionals concerned (GPP 10.1) (51).

## 4 Discussion

This first Tunisian CPG for ASD in children and adolescents represents a major step toward standardizing care and improving service delivery across health, education, and social systems. Developed using a rigorous ADAPTE methodology and supported by AGREE II appraisal, this guideline aligns international evidence-based recommendations with the specific organizational, cultural, and resource context of Tunisia.

In line with established international guidelines, including those developed by NICE (52), SIGN, ESCAP (53), and the American Academy of Pediatrics (AAP) (54), this guideline emphasizes early identification, comprehensive multidisciplinary assessment, and individualized intervention planning for children and adolescents with ASD. Similar to these guidelines, it highlights the importance of combining clinical judgment with standardized tools, promoting non-pharmacological interventions as first-line approaches, and addressing co-occurring conditions through a holistic framework. The contribution of the Tunisian guideline lies in endorsing and harmonizing evidence-based recommendations at the national level, while introducing targeted adaptations to the Tunisian healthcare and educational systems and establishing an implementation framework. The high proportion of recommendations adopted unchanged (85.8%) reflects rigorous appraisal of the source guideline and the retention of recommendations deemed applicable to the Tunisian context. Targeted adaptations addressed specific contextual considerations, including local resources, the availability of specialized professionals, medication availability and regulatory status, the applicability of screening instruments, the organization of primary and specialized care, and coordination with education and social services. This approach was intended to ensure feasibility while maintaining consistency with established international standards, which is particularly relevant in low- and middle-income settings.

A key strength of this guideline is its multidisciplinary and intersectoral approach. The involvement of child psychiatrists, pediatricians, neurologists, psychologists, speech and language therapists, occupational therapists, educators, and policy stakeholders, together with perspectives from the education sector and families obtained during external review, contributed to the clinical relevance and contextual applicability of the recommendations. This collaborative framework enhances the feasibility of implementation across different levels of care and promotes continuity between health, education, and social services, which is essential for ASD management.

The adaptation of the Singapore guideline as the primary source document ensured reliance on a recent, high-quality, and methodologically robust evidence base. The systematic use of AGREE II allowed transparent selection of guidelines, while the ADAPTE process ensured contextual relevance.

## 5 Limitations

Despite its methodological rigor, this guideline has some limitations. Participation of parent associations, school support assistants, and teachers in the drafting group remained limited. Although an autistic service user, caregivers, and a representative from the Ministry of Education contributed during the external review, people on the autism spectrum and self-advocates were not involved in the earlier stages of scope definition, recommendation formulation, or contextual adaptation. Their contributions helped contextualize the recommendations within the Tunisian educational system and identify implementation barriers, including limitations related to individualized educational plans and school support staff training. Future updates will aim to involve people on the autism spectrum, self-advocates, parents, caregivers, educators, and other relevant stakeholders from the early stages of guideline development.

In addition, the guideline was developed in French, the standard professional language used in Tunisian medical guidelines, which may limit accessibility for some families and non-medical stakeholders. To improve accessibility, simplified and Arabic-language versions have been developed. The French version is primarily intended for healthcare professionals involved in ASD care, while the simplified French and Arabic versions are intended for caregivers, teachers, primary care professionals, and other non-medical stakeholders. These versions will be disseminated through the STPEA and INEAS websites, professional training activities, and collaboration with educational and parent organizations.

Another limitation is that the supplementary evidence search supporting adapted and *de novo* recommendations ended in January 2025; therefore, publications after this cutoff were not incorporated into the current recommendations. This will be addressed through the planned guideline update cycle, with earlier revision if major new evidence emerges.

The use of a single primary source guideline represents another limitation. Although several international guidelines were reviewed during the selection process, the Singapore guideline was chosen to provide a coherent and structured basis for adaptation. As it was developed in a high-income healthcare setting, a systematic contextual adaptation process was undertaken to ensure the applicability of the recommendations to the Tunisian healthcare system.

The limited availability of published Tunisian epidemiological data and long-term outcome studies on autism spectrum disorder represents an important limitation. Consequently, some contextual information relied on grey literature, including academic dissertations and institutional reports, to complement the scarce peer-reviewed local evidence and better reflect the Tunisian healthcare context.

## 6 Implementation

The implementation of this guideline will require a coordinated national strategy involving professional societies, governmental health authorities, academic institutions, and primary care structures. The STPEA will play a central role in disseminating the guideline to relevant professional societies, government ministries, medical faculties, and primary healthcare professionals and in promoting its adoption through conferences, training activities, and collaboration with healthcare partners. Dissemination and initial training activities are planned within the first six months following publication, with broader integration into clinical services over the subsequent 18 months, leading up to the two-year evaluation. Training will initially prioritize primary care pediatricians and general practitioners, given their role in first-line screening, before being extended to other professionals involved in diagnosis and management, including psychologists, speech therapists, and educational staff. Implementation will also be supported by integration into existing child health services, the development of monitoring indicators, collaboration with stakeholders across the health and social sectors, and user-friendly formats, such as pocket versions, to facilitate access and uptake in routine practice.

A key facilitator for implementing the guideline is its contextual adaptation to the local Tunisian context, ensuring cultural relevance, practical feasibility, and alignment with existing health and education systems. This is further supported by the use of local research and the involvement of a multidisciplinary working group, which helps ensure recommendations are applicable across different sectors. Key challenges include limited resources and qualified personnel, geographic disparities in access to care, and variability in professionals’ familiarity with ASD screening and management recommendations. To mitigate these challenges, strategies such as decentralized training sessions and telemedicine-supported specialist consultations will be considered. Regional differences in implementation will be monitored to identify areas requiring additional training or support.

The STPEA will monitor the implementation of the recommendations and update the guideline every five years, or earlier if substantial new evidence or significant changes in clinical practice emerge. An initial evaluation will be conducted two years after publication through practice audits in healthcare facilities, focusing on the use of recommended screening tools, targeted questionnaires among professionals to assess knowledge, adherence and implementation, and stakeholder feedback. Key indicators will include the proportion of targeted professionals trained, the use of recommended screening tools, and adherence to key recommendations. These findings will be used to identify implementation gaps, guide corrective actions, and inform subsequent updates of the guideline.

## 7 Conclusion

The guideline recommends early developmental screening, standardized diagnostic assessment, multidisciplinary individualized intervention plans, and structured long-term follow-up within an integrated health, education, and social care framework. This first Tunisian ASD CPG aims to harmonize practices, improve the quality of care, and promote inclusion and optimal development of children and adolescents with ASD nationwide. Beyond its national scope, it may also support the adaptation of ASD guidelines in resource-variable settings and serve as a model for contextualizing international recommendations in low- and middle-income countries.

## References

[1] Lord C Brugha TS Charman T Cusack J Dumas G Frazier T . Autism spectrum disorder. Nat Rev Dis Primers. (2020) 6:5. doi: 10.1038/s41572-019-0138-4 31949163 PMC8900942

[2] World Health Organization . Autism Spectrum Disorders: Key Facts (2023). Available online at: https://www.who.int/news-room/fact-sheets/detail/autism-spectrum-disorders (Accessed May 6, 2026).

[3] Zeidan J Fombonne E Scorah J Ibrahim A Durkin MS Saxena S . Global prevalence of autism: a systematic review update. Autism Res. (2022) 15:778–90. doi: 10.1002/aur.2696 35238171 PMC9310578

[4] Manaa R . Étude De Prévalence Du Trouble Du Spectre De L'autisme Dans Une Population Du Centre tunisien dissertation. Monastir: Université de Monastir (2023).

[5] Ouali U Aissa A Larnaout A Abbes Z Charfi F Bouden A . Youth mental health in Tunisia: challenges and resources. Lancet Psychiatry. (2025) 12:96–7. doi: 10.1016/S2215-0366(24)00329-8 39521007 PMC12802523

[6] Fervers B Burgers JS Voellinger R Brouwers M Browman GP Graham ID . Guideline adaptation: an approach to enhance efficiency in guideline development and improve utilisation. BMJ Qual Saf. (2011) 20:228–36. doi: 10.1136/bmjqs.2010.043257 21209134

[7] Page MJ McKenzie JE Bossuyt PM Boutron I Hoffmann TC Mulrow CD . The PRISMA 2020 statement: an updated guideline for reporting systematic reviews. BMJ. (2021) 372:n71. doi: 10.1136/bmj.n71 33782057 PMC8005924

[8] Wong CM Aljunied M Chan DKL Cheong JMY Chew B Chin CH . 2023 clinical practice guidelines on autism spectrum disorder in children and adolescents in Singapore. Ann Acad Med Singap. (2024) 53:541–52. doi: 10.47102/annals-acadmedsg.2023307 38920181

[9] Landa RJ Kalb LG . Long-term outcomes of toddlers with autism spectrum disorders exposed to short-term intervention. Pediatrics. (2012) 130:S186–90. doi: 10.1542/peds.2012-0900Q 23118250

[10] Zwaigenbaum L Bauman ML Choueiri R Kasari C Carter A Granpeesheh D . Early intervention for children with autism spectrum disorder under 3 years of age: recommendations for practice and research. Pediatrics. (2015) 136:S60–81. doi: 10.1542/peds.2014-3667E 26430170 PMC9923898

[11] Barger BD Campbell JM McDonough JD . Prevalence and onset of regression within autism spectrum disorders: a meta-analytic review. J Autism Dev Disord. (2013) 43:817–28. doi: 10.1007/s10803-012-1621-x 22855372

[12] Hansen SN Schendel DE Francis RW Windham GC Bresnahan M Levine SZ . Recurrence risk of autism in siblings and cousins: a multinational, population-based study. J Am Acad Child Adolesc Psychiatry. (2019) 58:866–75. doi: 10.1016/j.jaac.2018.11.017 30851399 PMC6708733

[13] Slama S Bahia W Soltani I Gaddour N Ferchichi S . Risk factors in autism spectrum disorder: a Tunisian case-control study. Saudi J Biol Sci. (2022) 29:2749–55. doi: 10.1016/j.sjbs.2021.12.059 35531179 PMC9072901

[14] Barbaro J Sadka N Gilbert M Beattie E Li X Ridgway L . Diagnostic accuracy of the Social Attention and Communication Surveillance-Revised with Preschool tool for early autism detection in very young children. JAMA Netw Open. (2022) 5:e2146415. doi: 10.1001/jamanetworkopen.2021.46415 35275169 PMC8917423

[15] Lipkin PH Macias MM Council on Children with Disabilities Section on Developmental and Behavioral Pediatrics . Promoting optimal development: identifying infants and young children with developmental disorders through developmental surveillance and screening. Pediatrics. (2020) 145:e20193449. doi: 10.1542/peds.2019-3449 31843861

[16] Qiu S Qiu Y Li Y Cong X . Genetics of autism spectrum disorder: an umbrella review of systematic reviews and meta-analyses. Transl Psychiatry. (2022) 12:249. doi: 10.1038/s41398-022-02009-6 35705542 PMC9200752

[17] Huguet G Benabou M Bourgeron T . The genetics of autism spectrum disorders. In: Sassone-Corsi P Christen Y , editors. A Time for Metabolism and Hormones. Springer, Cham (2016). 28892342

[18] Patel R Sushko K van den Anker J Samiee-Zafarghandy S . Long-term safety of prenatal and neonatal exposure to paracetamol: a systematic review. Int J Environ Res Public Health. (2022) 19:2128. doi: 10.3390/ijerph19042128 35206317 PMC8871754

[19] DeStefano F Shimabukuro TT . The MMR vaccine and autism. Annu Rev Virol. (2019) 6:585–600. doi: 10.1146/annurev-virology-092818-015515 30986133 PMC6768751

[20] Christensen J Grønborg TK Sørensen MJ Schendel D Parner ET Pedersen LH . Prenatal valproate exposure and risk of autism spectrum disorders and childhood autism. JAMA. (2013) 309:1696–703. doi: 10.1001/jama.2013.2270 23613074 PMC4511955

[21] Sandbank M Bottema-Beutel K Crowley S Cassidy M Dunham K Feldman JI . Project AIM: autism intervention meta-analysis for studies of young children. Psychol Bull. (2020) 146:1–29. doi: 10.1037/bul0000215 31763860 PMC8783568

[22] White EN Ayres KM Snyder SK Cagliani RR Ledford JR . Augmentative and alternative communication and speech production for individuals with ASD: a systematic review. J Autism Dev Disord. (2021) 51:4199–212. doi: 10.1007/s10803-021-04868-2 33511525

[23] Asta L Persico AM . Differential predictors of response to Early Start Denver Model vs. Early Intensive Behavioral Intervention in young children with autism spectrum disorder: a systematic review and meta-analysis. Brain Sci. (2022) 12:1499. doi: 10.3390/brainsci12111499 36358426 PMC9688546

[24] Reichow B Hume K Barton EE Boyd BA . Early intensive behavioral intervention (EIBI) for young children with autism spectrum disorders (ASD). Cochrane Database Syst Rev. (2018) 5:CD009260. doi: 10.1002/14651858.CD009260.pub3 29742275 PMC6494600

[25] Schreibman L Dawson G Stahmer AC Landa R Rogers SJ McGee GG . Naturalistic developmental behavioral interventions: empirically validated treatments for autism spectrum disorder. J Autism Dev Disord. (2015) 45:2411–28. doi: 10.1007/s10803-015-2407-8 25737021 PMC4513196

[26] Berggren S Fletcher-Watson S Milenkovic N Marschik PB Bölte S Jonsson U . Emotion recognition training in autism spectrum disorder: a systematic review of challenges related to generalizability. Dev Neurorehabil. (2018) 21:141–54. doi: 10.1080/17518423.2017.1305004 28394669

[27] Hume K Steinbrenner JR Odom SL Morin KL Nowell SW Tomaszewski B . Evidence-based practices for children, youth, and young adults with autism: third generation review. J Autism Dev Disord. (2021) 51:4013–32. doi: 10.1007/s10803-020-04844-2 33449225 PMC8510990

[28] Chan PLC Poon MYC Bux V Wong SKF Chu AWY Louie FTM . Occupational therapy using an Ayres sensory integration approach for school-age children: a randomized controlled trial. World Fed Occup Ther Bull. (2023) 79:228–35.

[29] Bodison SC Parham LD . Specific sensory techniques and sensory environmental modifications for children and youth with sensory integration difficulties: a systematic review. Am J Occup Ther. (2018) 72:7201190040p1–11. doi: 10.5014/ajot.2018.029413 29280714

[30] Gosling CJ Cartigny A Mellier BC Solanes A Radua J Delorme R . Efficacy of psychosocial interventions for autism spectrum disorder: an umbrella review. Mol Psychiatry. (2022) 27:3647–56. doi: 10.1038/s41380-022-01670-z 35790873 PMC9708596

[31] Sturman N Deckx L van Driel ML . Methylphenidate for children and adolescents with autism spectrum disorder. Cochrane Database Syst Rev. (2017) 11:CD011144. doi: 10.1002/14651858.CD011144.pub2 29159857 PMC6486133

[32] Mano-Sousa BJ Pedrosa AM Alves BC Galduróz JCF Belo VS Chaves VE . Effects of risperidone in autistic children and young adults: a systematic review and meta-analysis. Curr Neuropharmacol. (2021) 19:538–52. doi: 10.2174/1570159X18666200529151741 32469700 PMC8206457

[33] Rossignol DA Frye RE . Melatonin in autism spectrum disorders: a systematic review and meta-analysis. Dev Med Child Neurol. (2011) 53:783–92. doi: 10.1111/j.1469-8749.2011.03980.x 21518346

[34] Williams K Brignell A Randall M Silove N Hazell P . Selective serotonin reuptake inhibitors (SSRIs) for autism spectrum disorders (ASD). Cochrane Database Syst Rev. (2013) 2013:CD004677. doi: 10.1002/14651858.CD004677.pub3 23959778 PMC11990048

[35] Hurwitz R Blackmore R Hazell P Williams K Woolfenden S . Tricyclic antidepressants for autism spectrum disorders (ASD) in children and adolescents. Cochrane Database Syst Rev. (2012) 2012:CD008372. doi: 10.1002/14651858.CD008372.pub2 22419332 PMC12053881

[36] Hirota T Veenstra-Vanderweele J Hollander E Kishi T . Antiepileptic medications in autism spectrum disorder: a systematic review and meta-analysis. J Autism Dev Disord. (2014) 44:948–57. doi: 10.1007/s10803-013-1952-2 24077782

[37] Smida S . Inclusion Scolaire Des Enfants Avec Trouble Du Spectre De L'autisme Au Gouvernorat De Monastir: État des lieux dissertation. Monastir: Université de Monastir (2024).

[38] Organization for Autism Research . Factors Influencing the Educational Placement of Students With Autism Spectrum Disorders (2016). Available online at: https://researchautism.org/research-item/factors-influencing-the-educational-placement-of-students-with-autism-spectrum-disorders/ (Accessed May 7, 2026).

[39] Pancheva R Chamova R Marinov D Toneva A Dzhogova M Eyubova S . Therapeutic diets and supplementation: exploring their impact on autism spectrum disorders in childhood - a narrative review of recent clinical trials. Res Autism Spectr Disord. (2024) 112:102352. doi: 10.1016/j.rasd.2024.10235242574925

[40] Podgórska-Bednarz J Perenc L . Hyperbaric oxygen therapy for children and youth with autism spectrum disorder: a review. Brain Sci. (2021) 11:916. doi: 10.3390/brainsci11070916 34356150 PMC8303909

[41] Paprocka J Kaminiów K Kozak S Sztuba K Emich-Widera E . Stem cell therapies for cerebral palsy and autism spectrum disorder - a systematic review. Brain Sci. (2021) 11:1606. doi: 10.3390/brainsci11121606 34942908 PMC8699362

[42] Morris P Hope E Foulsham T Mills JP . Dancing out for a voice: a narrative review of the literature exploring autism, physical activity, and dance. J Bodyw Mov Ther. (2023) 33:202–15. doi: 10.1016/j.jbmt.2022.09.016 36775520

[43] Vogel SW Mullins KL Kumar S . Art therapy for children and adolescents with autism: a systematic review. Int J Art Ther. (2025) 30:113–22. doi: 10.1080/17454832.2024.234337337339054

[44] Applewhite B Cankaya Z Heiderscheit A Himmerich H . A systematic review of scientific studies on the effects of music in people with or at risk for autism spectrum disorder. Int J Environ Res Public Health. (2022) 19:5150. doi: 10.3390/ijerph19095150 35564544 PMC9100336

[45] Geretsegger M Fusar-Poli L Elefant C Mössler KA Vitale G Gold C . Music therapy for autistic people. Cochrane Database Syst Rev. (2022) 2022:CD004381. doi: 10.1002/14651858.CD004381.pub4 35532041 PMC9082683

[46] Bougeard C Picarel-Blanchot F Schmid R Campbell R Buitelaar J . Prevalence of autism spectrum disorder and co-morbidities in children and adolescents: a systematic literature review. Front Psychiatry. (2021) 12:744709. doi: 10.3389/fpsyt.2021.744709 34777048 PMC8579007

[47] Khachadourian V Mahjani B Sandin S Kolevzon A Buxbaum JD Reichenberg A . Comorbidities in autism spectrum disorder and their etiologies. Transl Psychiatry. (2023) 13:71. doi: 10.1038/s41398-023-02374-w 36841830 PMC9958310

[48] Kirby AV Baranek GT Fox L . Longitudinal predictors of outcomes for adults with autism spectrum disorder: systematic review. OTJR (Thorofare N J). (2016) 36:55–64. doi: 10.1177/1539449216650182 27504878

[49] Woodman AC Mailick MR Greenberg JS . Trajectories of internalizing and externalizing symptoms among adults with autism spectrum disorders. Dev Psychopathol. (2016) 28:565–81. doi: 10.1017/S095457941500108X 26612272 PMC4828272

[50] Dawson-Squibb JJ Davids EL Harrison AJ Molony MA de Vries PJ . Parent education and training for autism spectrum disorders: scoping the evidence. Autism. (2020) 24:7–25. doi: 10.1177/1362361319841739 31070045

[51] Hurt L Langley K North K Southern A Copeland L Gillard J . Understanding and improving the care pathway for children with autism. Int J Health Care Qual Assur. (2019) 32:208–23. doi: 10.1108/IJHCQA-08-2017-0153 30859873

[52] National Institute for Health and Care Excellence (NICE) . 2021 Surveillance of Autism (NICE Guidelines CG128, CG142 and CG170) (2021). Available online at: http://www.ncbi.nlm.nih.gov/books/NBK571333/ (Accessed May 6, 2026). 34165941

[53] Fuentes J Hervás A Howlin P ESCAP ASD Working Party . ESCAP practice guidance for autism: a summary of evidence-based recommendations for diagnosis and treatment. Eur Child Adolesc Psychiatry. (2021) 30:961–84. doi: 10.1007/s00787-020-01587-4 32666205 PMC8140956

[54] Hyman SL Levy SE Myers SM Council on Children with Disabilities Section on Developmental and Behavioral Pediatrics . Identification, evaluation, and management of children with autism spectrum disorder. Pediatrics. (2020) 145:e20193447. doi: 10.1542/peds.2019-3447 31843864

