# Supplementary material for: Development and contextual adaptation of the first national clinical practice guideline for autism spectrum disorder in children and adolescents in Tunisia
Source: Front Psychiatry. 2026 Oct 20;17:1926851. doi: 10.3389/fpsyt.2026.1926851 (PMC13727530; doi:10.3389/fpsyt.2026.1926851)
Supplement: Supplementary file 1 [file Table1.docx]

**Title: Development and contextual adaptation of the first national clinical practice guideline for autism spectrum disorder in children and adolescents in Tunisia**

Randaline Ayoub^1^**^,2*^**, Hela Ben Abid^1,2^, Soumaya Bourgou^3,4^, Imen Hadj Kacem^5^,  Soumeyya Halayem^6^, Khaoula Khemekhem^5^, Fatma Charfi^3,4^, Asma Bouden^6^

**Randaline Ayoub, MD (Corresponding author)**

Assistant Professor in Child and Adolescent Psychiatry, Fattouma Bourguiba University Hospital, Faculty of Medicine, University of Monastir, Monastir, Tunisia

Permanent Full poster address: Faculty of Medicine, University of Monastir, Avicenne Avenue, Monastir 5000, Tunisia

Phone: +216 21 481 979; Fax: +216 73 460 309

E-mail address: [ayoub.randaline@gmail.com](mailto:ayoub.randaline@gmail.com)

Supplementary Table S1: Literature search strategy and results

| Source | Search date | Search strategy/keywords | Publication period | Records retrieved |
| --- | --- | --- | --- | --- |
| Pubmed | March 2024 | (“autism” OR “autism spectrum disorder” OR “autistic disorder” OR “infantile autism” OR “childhood autism”) AND (“practice guidelines” OR “clinical practice guidelines” OR “evidence-based medicine”); filter: Practice Guideline, English, french | 2015-2024 | 4 |
| Trip Database | March 2024 | autism spectrum disorder AND clinical guideline OR evidence-based medicine | 2015-2024 | 249 |
| Embase | March 2024 | autism spectrum disorder | 2015-2024 | 15 |
| GIN | March 2024 | autism | 2015-2024 | 11 |
| NICE | March 2024 | autism spectrum disorders | 2015-2024 | 10 |
| SIGN | March 2024 | autism | 2015-2024 | 2 |
| IACAPAP | March 2024 | autism guidelines | 2015-2024 | 0 |
| ESCAP | March 2024 | Autism guidelines | 2015-2024 | 4 |
