# Supplementary material for: Development and contextual adaptation of the first national clinical practice guideline for autism spectrum disorder in children and adolescents in Tunisia
Source: Front Psychiatry. 2026 Oct 20;17:1926851. doi: 10.3389/fpsyt.2026.1926851 (PMC13727530; doi:10.3389/fpsyt.2026.1926851)
Supplement: Supplementary file 2 [file Table2.docx]

**Title: Development and contextual adaptation of the first national clinical practice guideline for autism spectrum disorder in children and adolescents in Tunisia**

Randaline Ayoub^1^**^,2*^**, Hela Ben Abid^1,2^, Soumaya Bourgou^3,4^, Imen Hadj Kacem^5^,  Soumeyya Halayem^6^, Khaoula Khemekhem^5^, Fatma Charfi^3,4^, Asma Bouden^6^

**Randaline Ayoub, MD (Corresponding author)**

Assistant Professor in Child and Adolescent Psychiatry, Fattouma Bourguiba University Hospital, Faculty of Medicine, University of Monastir, Monastir, Tunisia

Permanent Full poster address: Faculty of Medicine, University of Monastir, Avicenne Avenue, Monastir 5000, Tunisia

Phone: +216 21 481 979; Fax: +216 73 460 309

E-mail address: [ayoub.randaline@gmail.com](mailto:ayoub.randaline@gmail.com)

**Supplementary Table S2: Guidelines assessed in full text and reasons for exclusion based on PIPOH criteria**

| **Guideline** | **Status** | **PIPOH criterion not met** | **Justification** |
| --- | --- | --- | --- |
| NICE | Included |  |  |
| Singapore | Included |  |  |
| Australian | Included |  |  |
| SIGN | Included |  |  |
| ESCAP | Included |  |  |
| New Zealand | Excluded | I , H | **I:** Scope of interventions extends beyond the clinical management of ASD.  **H:** Scope extends beyond healthcare settings to a broader multisectoral and society-wide framework. |
| Spain | Excluded | I , H | **I incomplete:** no therapeutic recommendations  **H restricted:** primary care only |
| Italy | Excluded | I | Does not cover screening or follow-up |
| HAS | Excluded | P | Addresses adults with autism spectrum disorder |
| Korea | Excluded | I | No recommendations on screening/diagnosis/treatment/follow-up |
| American academy of pediatrics (AAP) | Excluded | H | Restricted to ambulatory primary pediatric care within the medical home model. |
