# Supplementary material for: Development and contextual adaptation of the first national clinical practice guideline for autism spectrum disorder in children and adolescents in Tunisia
Source: Front Psychiatry. 2026 Oct 20;17:1926851. doi: 10.3389/fpsyt.2026.1926851 (PMC13727530; doi:10.3389/fpsyt.2026.1926851)
Supplement: Supplementary file 3 [file Table3.docx]

**Title: Development and contextual adaptation of the first national clinical practice guideline for autism spectrum disorder in children and adolescents in Tunisia**

Randaline Ayoub^1^**^,2*^**, Hela Ben Abid^1,2^, Soumaya Bourgou^3,4^, Imen Hadj Kacem^5^,  Soumeyya Halayem^6^, Khaoula Khemekhem^5^, Fatma Charfi^3,4^, Asma Bouden^6^

**Randaline Ayoub, MD (Corresponding author)**

Assistant Professor in Child and Adolescent Psychiatry, Fattouma Bourguiba University Hospital, Faculty of Medicine, University of Monastir, Monastir, Tunisia

Permanent Full poster address: Faculty of Medicine, University of Monastir, Avicenne Avenue, Monastir 5000, Tunisia

Phone: +216 21 481 979; Fax: +216 73 460 309

E-mail address: [ayoub.randaline@gmail.com](mailto:ayoub.randaline@gmail.com)

**Supplementary Table S 3:**  Representative examples of recommendations adopted, adapted, or developed *de novo* during the adaptation of the Singapore Clinical Practice Guideline to the Tunisian context.

| Singapore guideline recommendation | Tunisian recommendation | Adaptation decision | Rationale for adaptation | Expected implementation impact |
| --- | --- | --- | --- | --- |
| Atomoxetine may be considered for managing attention deficit hyperactivity disorder in children and adolescents on the autism spectrum if methylphenidate has been tried unsuccessfully or is contraindicated or not tolerated. | The recommendation was not retained. The guideline states that atomoxetine may represent a therapeutic alternative; however, it is currently not commercially available in Tunisia. | Adapted | National drug availability differs from Singapore. | Prevents recommending a treatment that is currently unavailable while informing clinicians about internationally accepted therapeutic alternatives. |
| Risperidone and aripiprazole can be used for challenging behaviours (irritability and hyperactivity) in children and adolescents on the autism spectrum in the short term. | Risperidone may be used for the short-term management of challenging behaviours (irritability and hyperactivity) in children and adolescents with autism spectrum disorder. There is insufficient evidence to support long-term use (>6 months). | Adapted | Aripiprazole does not have national marketing authorization (AMM) for this indication in Tunisia. | Aligns pharmacological recommendations with the national regulatory framework and prescribing practice. |
| Social skills intervention is recommended for children and adolescents on the autism spectrum to improve social communication and interaction skills. | Social skills intervention is recommended for children and adolescents with autism spectrum disorder to improve social communication and interaction skills. It should be tailored to the individual's needs, preferences and environment. | Adopted | Strong evidence and direct applicability to the Tunisian context. | Supports immediate implementation within multidisciplinary rehabilitation services. |
| Caregiver education and training programmes should be incorporated into intervention programmes whenever possible. | Parent and caregiver education and training should be integrated into intervention programmes for children and adolescents with autism spectrum disorder whenever possible. | Adopted | Strong evidence and direct applicability to the Tunisian context. | Reinforces family-centred care and promotes caregiver involvement throughout the intervention process. |
| Augmentative and Alternative Communication (AAC) may be used for children and adolescents on the autism spectrum to support communicative understanding and expression. | Augmentative and Alternative Communication (AAC) may be used for children and adolescents with autism spectrum disorder to support communication, both in comprehension and expression. AAC should be individualized according to the person's communication needs, preferences and environment. | Adopted | Recommendation considered applicable in Tunisia. | Promotes individualized communication support across healthcare and educational settings. |
| Parents and caregivers should consult appropriate professionals when considering educational interventions and school placement for their child on the autism spectrum, such as professionals familiar with the educational provisions in Singapore. | Parents and caregivers should consult qualified professionals, including child psychiatrists, educational psychologists and regional rehabilitation teams, when considering educational interventions and school inclusion for their child with autism spectrum disorder. | Adapted | Adapted to reflect the organization of the Tunisian healthcare and educational systems. | Improves coordination between healthcare professionals, families and educational services. |
|  | All schools in Tunisia are inclusive. Inclusive education should provide appropriate educational accommodations, including reduced class sizes where available and adapted teaching and assessment measures, in accordance with the Tunisian educational system. | De novo | Developed by multidisciplinary expert consensus to reflect the Tunisian legal and educational framework for inclusive education. | Supports implementation of national inclusive education policies and strengthens collaboration between the health and education sectors. |

*The examples presented were selected to illustrate the different types of contextual adaptations undertaken during the ADAPTE process and do not represent the complete list of recommendations included in the guideline.
