# Supplementary material for: Development and contextual adaptation of the first national clinical practice guideline for autism spectrum disorder in children and adolescents in Tunisia
Source: Front Psychiatry. 2026 Oct 20;17:1926851. doi: 10.3389/fpsyt.2026.1926851 (PMC13727530; doi:10.3389/fpsyt.2026.1926851)
Supplement: Supplementary file 4 [file Table4.docx]

**Title: Development and contextual adaptation of the first national clinical practice guideline for autism spectrum disorder in children and adolescents in Tunisia**

Randaline Ayoub^1^**^,2*^**, Hela Ben Abid^1,2^, Soumaya Bourgou^3,4^, Imen Hadj Kacem^5^,  Soumeyya Halayem^6^, Khaoula Khemekhem^5^, Fatma Charfi^3,4^, Asma Bouden^6^

**Randaline Ayoub, MD (Corresponding author)**

Assistant Professor in Child and Adolescent Psychiatry, Fattouma Bourguiba University Hospital, Faculty of Medicine, University of Monastir, Monastir, Tunisia

Permanent Full poster address: Faculty of Medicine, University of Monastir, Avicenne Avenue, Monastir 5000, Tunisia

Phone: +216 21 481 979; Fax: +216 73 460 309

E-mail address: [ayoub.randaline@gmail.com](mailto:ayoub.randaline@gmail.com)

Supplementary File S4: Recommendations

R= Recommendation, GPP= Good Practice Point

1. ***Screening and diagnosis***

**GPP 1.1:** Professionals should identify autism at an early stage to allow for early referral and intervention, which can lead to better long-term outcomes.

**GPP 1.2:** Surveillance for early signs of autism should be integrated into a national developmental surveillance programme.

**GPP 1.3:** Developmental surveillance should be carried out repeatedly and at regular intervals to detect signs of autism.

**GPP 1.4:** Concerns raised by parents or caregivers regarding the child's communication, social interaction, play and behaviour should be elicited at every healthcare visit. Parents should be encouraged to inform health professionals if their child shows difficulties in these areas.

**GPP 1.5:** Concerns raised by preschool teachers regarding a child's communication, social interaction, play and behaviour should be taken into account as part of developmental surveillance programmes for preschool-age children.

**GPP 1.6:** Professionals should promptly refer preschool-age children with communication, social interaction, play or behavioural concerns to specialists, rather than reassuring parents or adopting a wait-and-see approach.

**GPP 1.7:** Children presenting with one or more of the following clinical features should be promptly referred for a comprehensive developmental assessment:

• Any regression or loss of language or social skills.

• Absence of babbling, use of social gestures (waving, saying "bye-bye", pointing), shared enjoyment (spontaneous showing, following pointing/gaze), or response to their name being called by around 12 months.

• Absence of single meaningful words, absence of following instructions, or absence of pretend play by 18 months.

• Lack of eye contact or social responsiveness, or any unusual, repetitive, rigid or obsessive behaviour, or sensory symptoms of hypo/hyper-sensitivity, at any age.

**GPP 1.8:** Professionals should remain alert to the possibility of autism in any child or adolescent presenting with persistent difficulties related to communication, social interaction, behaviour or mental health.

**GPP 1.9:** Health professionals should be aware of the factors associated with an increased likelihood of developing autism and may consider targeted screening for children presenting with developmental concerns or these factors. Specific factors associated with an increased likelihood of autism include:

• A history of autism in a sibling.

• Prematurity < 35 weeks of gestation or birth weight < 2500 g.

• Neonatal hypoxic encephalopathy.

• A genetic syndrome known to be associated with autism.

• In-utero exposure to maternal antiepileptic medication.

• Advanced parental age at the child's birth (> 40 years).

• A parental history of mental health disorders.

**R 1.10:** Based on current evidence, the routine use of autism-specific screening tools in the general paediatric population with no risk factors is not recommended.

**R 1.11:** When a developmental delay is suspected in a child, the use of an autism-specific screening tool may complement the clinical judgement of health professionals but should not replace it. Such a tool alone cannot confirm or rule out a diagnosis of autism.

**GPP 1.12:** Health professionals who decide to use an autism screening tool should be familiar with its performance characteristics (e.g., false positives, false negatives) and its limitations. They should also bear in mind that performance characteristics may vary across cultures and contexts.

**R 1.13:** Autism-specific screening tools must be used within the age range for which they have been validated. Health professionals should know that screening tools have proven more accurate for children aged 21 months and older than for those aged 12 to 20 months.

**GPP 1.14:** Although research has identified differences in neurophysiological biomarkers between children with autism spectrum disorder and neurotypical children, these biomarkers are not sufficiently developed to serve as accurate and reliable autism screening tools. Based on current evidence, neurophysiological biomarkers and other isolated markers are not recommended for routine clinical use in autism screening.

**GPP 1.15:** Professionals involved in diagnosing autism in children and adolescents should use the most recent version of the DSM or ICD, and should specify which classification system was used.

**GPP 1.16:** Professionals involved in the diagnostic assessment of autism spectrum disorder should be aware that some children may no longer meet DSM-5-TR diagnostic criteria despite having met DSM-IV-TR criteria. Some of these children may meet criteria for social communication disorder and may require interventions similar to those used for autism spectrum disorder.

**GPP 1.17:** Children assessed for autism spectrum disorder should undergo a medical examination in order to guide any necessary physical investigations and to offer additional medical treatment if needed.

**GPP 1.18:** A multidisciplinary approach is recommended wherever possible for diagnosing autism spectrum disorder in children and adolescents, particularly in complex cases or where the clinician judges that high diagnostic reliability can only be achieved through a holistic multidisciplinary assessment.

**GPP 1.19:** A single-clinician approach to diagnosing autism spectrum disorder may be considered when the following conditions are met:

• The diagnostic process is conducted by physicians or psychologists with adequate training and experience in diagnosing autism in children and adolescents (Table 1.6).

• Information from multiple sources and different contexts is available, to obtain a comprehensive overall picture of the child.

• Direct observation of, and interaction with, the child is possible.

• Recent, comprehensive documentation of the child's autism symptoms is available, meeting current international diagnostic criteria for autism spectrum disorder (e.g., DSM-5-TR).

**R 1.20:** Assessment and diagnosis should not rely solely on autism-specific diagnostic instruments. They must build a holistic profile of the child, including developmental, medical and social history, a neurological and physical examination, consideration of differential diagnoses and comorbid, cognitive, sensory and academic conditions, as well as the behavioural and adaptive profile. Likewise, strengths, skills and needs must be assessed to guide the therapeutic plan. Autism-specific diagnostic instruments can complement the assessment process, clinical observation and information gathering.

**GPP 1.21:** Information gathered to establish a diagnosis should include reports or observations of the child in the family setting, in the community, or outside the clinical setting.

**GPP 1.22:** Professionals involved in the diagnostic process should consider that females with autism spectrum disorder may present a different clinical profile and level of need compared with their male counterparts.

**GPP 1.23:** Professionals should take cultural differences into account when assessing a person with autism. Understanding the impact of such cultural variation on the clinical presentation of the disorder will help professionals tailor their assessments to patients from different cultural backgrounds.

**GPP 1.24:** Professionals should be aware that the features of intellectual disability (ID) and autism overlap when assessing autism in people with intellectual disability. Autism should not be diagnosed if the symptoms are better explained by ID.

2. ***Etiology and diagnostic investigations***

**GPP 2.1:** Health professionals should be aware of the strong genetic heritability of autism and should systematically screen for the condition in siblings and first-degree relatives of individuals with autism spectrum disorder.

**GPP 2.2:** Health professionals should be aware that certain genetic conditions or syndromes may be associated with autism. They should therefore regularly monitor affected children for associated autistic symptoms. Genetic conditions or syndromes frequently associated with autism include fragile X syndrome, Angelman syndrome, tuberous sclerosis, Rett syndrome, PTEN hamartoma tumour syndrome, and trisomy 21.

**GPP 2.3:** Health professionals should be aware that there is insufficient evidence of a link between maternal paracetamol use during pregnancy and an increased likelihood of autism in offspring. Pregnant women therefore do not need to avoid paracetamol during pregnancy when it is clinically indicated.

**GPP 2.4:** Health professionals should discuss the indications and side effects of the various antiepileptic medications. Maternal use of sodium valproate during pregnancy is associated with an increased risk of autism in offspring.

**GPP 2.5:** Health professionals should be aware that there is insufficient evidence of an association between epidural analgesia given to women during labour and an increased likelihood of autism in the child. Epidural analgesia should therefore not be avoided when it is deemed necessary.

**GPP 2.6:** Parents should be reassured that childhood vaccinations are not associated with autism, and should follow the recommended vaccination schedule. Health professionals should administer vaccinations to children according to the vaccination schedule, including the Measles, Mumps and Rubella (MMR) vaccine.

**GPP 2.7:** Routine screening tests measuring heavy metal levels (antimony, aluminium, arsenic, cadmium, lead, manganese, mercury, nickel, silver, thallium) are not recommended for children with autism spectrum disorder. No causal relationship has been identified.

**GPP 2.8:** Health professionals may consider testing for mercury toxicity in certain children with autism spectrum disorder who present with severe neurological and immunological problems.

**GPP 2.9:** Health professionals may consider testing for lead toxicity in certain children with autism spectrum disorder in whom an associated pica-type eating disorder is suspected.

**GPP 2.10:** Professionals should be aware of the negative effects of screen exposure on children with autism.

**GPP 2.11:** Children presenting with autism spectrum disorder together with clinical features suggestive of an underlying genetic condition (such as microcephaly, seizures, dysmorphic features, congenital anomalies, or a family history of developmental disorders) should be referred to a clinical geneticist for diagnostic confirmation and genetic counselling. Examples of such genetic conditions or syndromes include fragile X syndrome, Angelman syndrome, tuberous sclerosis, Rett syndrome, PTEN hamartoma tumour syndrome, and trisomy 21.

**GPP 2.12:** Children diagnosed with autism may benefit from genetic testing when indicated by a physician. The discussion regarding which genetic test(s) to consider should be led by a clinical geneticist.

**GPP 2.13:** Brain magnetic resonance imaging (MRI) with spectroscopy should not be a routine investigation in isolated autism spectrum disorder. It should, however, be offered to children with autism who have an abnormal head circumference, psychomotor regression, global developmental delay, epilepsy, or a focal neurological sign, in consultation with a paediatric neurologist.

**GPP 2.14:** Electroencephalography (EEG) should be offered to certain children with autism spectrum disorder who have clinically confirmed seizures, features suggestive of seizures, and/or cognitive regression. This investigation should only be requested after a neurological examination and in consultation with a paediatric neurologist.

**GPP 2.15:** A metabolic work-up should be indicated in children with syndromic autism spectrum disorder presenting with the following clinical features: dysmorphic features, ataxia, microcephaly, epilepsy, severe intellectual disability, cyclical vomiting, or familial consanguinity. If autism spectrum disorder remains without an evident aetiology, a minimal metabolic work-up targeting treatable conditions should be considered. This work-up includes ammonia and uric acid levels, a Guthrie test with phenylalanine measurement, amino acid chromatography, homocysteine measurement, organic acid chromatography, and urinary guanidinoacetate and creatine/creatinine ratio measurement.

**GPP 2.16:** Routine stool analysis for yeast or microbiota is not recommended in children with autism spectrum disorder.

3. ***Intervention***

**R 3.1:** Augmentative and Alternative Communication (AAC) may be used for children and adolescents with autism spectrum disorder to support communication, both in terms of comprehension and expression. AAC should be tailored to each individual's needs, communication style, preferences and environment.

**R 3.2:** Communication-based interventions (speech and language therapy, pivotal response training) may be used in children and adolescents with autism spectrum disorder, as they improve social communication outcomes (including joint attention, social engagement and initiation) and can improve receptive language, expressive language and speech prosody.

**R 3.3:** Developmental interventions (implemented according to a developmental sequence and focused on supporting children's skill-learning through interactions with others, particularly parents or caregivers) may be used for children and adolescents with autism spectrum disorder to improve communication and social interaction difficulties.

**R 3.4:** Early Intensive Behavioural Intervention (EIBI) may be considered as a technique that improves the development of adaptive skills and cognitive abilities in children with autism spectrum disorder. It should be delivered by qualified professionals and at sufficient intensity, and should be based on pre-established goals for the child and family.

**R 3.5:** Emotional Regulation Therapy (ERT) involves treatment modalities (computer programmes, videos, games) to teach the recognition, perception and management of emotions in children and adolescents with autism spectrum disorder, using a social-pragmatic approach. An ERT-based intervention may be considered a therapy that improves emotion recognition and socio-communicative skills in children and adolescents with autism spectrum disorder.

**R 3.6:** Naturalistic Developmental Behavioural Interventions (NDBI), which are intervention practices integrating behavioural and developmental theories (delivered in natural environments and using child-centred, motivation-based strategies to teach developmentally appropriate skills within play and routine activities), may be offered to children with autism spectrum disorder to improve social communication, language, cognitive and play skills.

**R 3.7:** Play-based intervention may involve the use of a variety of materials such as games, toys and activities to promote play skills and social communication, whereas play therapy is a non-directive approach aimed at addressing emotional and behavioural difficulties. These approaches may be used in children with autism spectrum disorder to improve language, joint attention and social engagement skills, particularly for those under 12 years of age.

**R 3.8:** Sensory integration therapy incorporating elements described by Ayres may be recommended as a therapeutic intervention for children with autism spectrum disorder aged 3 to 12 years, to improve social communication and functional participation.

**R 3.9:** Modifications to the sensory environment and sensory modulation strategies may be considered for certain children and adolescents with autism spectrum disorder to address their specific sensory needs.

**R 3.10:** Weighted vests are not recommended as a therapeutic intervention for children and adolescents with autism spectrum disorder, due to insufficient evidence regarding their benefits and risks.

**R 3.11:** Social skills intervention is recommended for children and adolescents with autism spectrum disorder to improve their social communication and interaction skills. It may also have positive effects on challenging behaviours, adaptive and cognitive abilities, and academic and learning skills. Social skills intervention should be tailored to the individual's needs, preferences and environment.

**R 3.12:** Visual supports (pictures, objects, written words, lists, schedules, choice boards) should be used for children and adolescents with autism spectrum disorder.

**R 3.13:** CBT may be offered to children and adolescents with autism spectrum disorder who have sufficient verbal and reasoning abilities to address emotion-related difficulties such as anxiety and anger. Modifications may be needed to facilitate understanding and application of CBT strategies in this population. Involving parents and caregivers can support the generalisation of strategies in younger children.

**GPP 3.14:** Cognitive Remediation Therapy (CRT) may be offered to children/adolescents with autism spectrum disorder who are verbal and without moderate to severe intellectual disability, in order to improve their executive functions and academic learning. CRT should be delivered by qualified professionals at the recommended intensity. Training therapists in the use of CRT and involving parents in the remediation process should continue to be pursued and encouraged.

**GPP 3.15:** The NEAR method for cognitive remediation and social skills training could be offered to adolescents with autism spectrum disorder who are verbal and do not have moderate to severe intellectual disability, in order to improve their cognitive and social abilities.

4. ***Pharmacological treatment***

**R 4.1:** Methylphenidate should be considered the first-line treatment for ADHD in children and adolescents with autism spectrum disorder. Any therapeutic plan should include non-pharmacological management.

**R 4.2:** Risperidone may be used short-term to treat challenging behaviours (irritability and hyperactivity) in children and adolescents with autism spectrum disorder. There is insufficient evidence to conclude that risperidone is beneficial in the long term (more than 6 months). Risperidone may cause weight gain and drowsiness.

**R 4.3:** Selective serotonin reuptake inhibitors (SSRIs) (e.g., fluoxetine, sertraline, escitalopram, paroxetine) should not be used to treat the core symptoms of autism in children and adolescents. SSRIs may be used to treat co-occurring psychiatric disorders such as anxiety, depression or OCD. This indication should be made by a qualified specialist.

**R 4.4:** Tricyclic antidepressants (TCAs) such as clomipramine should not be used to manage challenging behaviours in children and adolescents with autism spectrum disorder. TCAs may be considered as a second- or third-line option for treating psychiatric disorders (e.g., depression) on the prescription of a qualified specialist.

**R 4.5:** Antiepileptics/mood stabilisers should not be routinely used to manage challenging behaviours in children and adolescents with ASD. They may be considered as a second- or third-line option for treating challenging behaviours or psychiatric disorders in children and adolescents with autism spectrum disorder, on the prescription of a qualified specialist.

**R 4.6:** Buspirone should not be used as an adjunct to risperidone for treating challenging behaviours in children and adolescents with autism spectrum disorder.

**R 4.7:** Celecoxib should not be used as an adjunct to risperidone in the management of challenging behaviours in autism spectrum disorder.

**R 4.8:** Galantamine should not be used in combination with risperidone to treat challenging behaviours in children and adolescents with autism spectrum disorder.

**R 4.9:** Melatonin may be considered for sleep disorders when psychosocial interventions have proven ineffective. Its prescription should be combined with a psychosocial intervention delivered by a specialist qualified in the assessment and management of sleep disorders in children and adolescents with autism spectrum disorder.

**R 4.10:** Acamprosate should not be used to treat the core symptoms of autism in children and adolescents.

**R 4.11:** Amantadine should not be used to treat the core symptoms of autism in children and adolescents. Amantadine should also not be used as an adjunct to risperidone for treating irritability in children and adolescents with autism spectrum disorder.

**R 4.12:** Arbaclofen should not be used to treat the core symptoms of autism in children and adolescents.

**R 4.13:** Based on current evidence, bumetanide should not be used to treat the core symptoms of autism in children and adolescents. New clinical trial results on bumetanide are awaited.

**R 4.14:** D-cycloserine should not be used to treat the core symptoms of autism in children and adolescents.

**R 4.15:** Memantine should not be used to treat the core symptoms of autism in children and adolescents. It should also not be used as an adjunct to risperidone for treating irritability in children and adolescents with autism spectrum disorder.

**R 4.16:** N-acetylcysteine should not be used in children and adolescents to treat the core symptoms of autism spectrum disorder. There is currently insufficient evidence regarding the use of N-acetylcysteine as an adjunct to risperidone for treating irritability in this condition and age group. Further studies are required.

**R 4.17:** Riluzole should not be used to treat the core symptoms of autism spectrum disorder in children and adolescents. It should also not be used as an adjunct to risperidone for treating irritability in children and adolescents with autism spectrum disorder.

**R 4.18:** Based on current evidence, intranasal oxytocin should not be used to treat the core symptoms of autism in children and adolescents. Further clinical trial results on oxytocin are awaited.

**R 4.20:** Insulin-like growth factor 1 (IGF-1; e.g., trofinetide, mecasermin) should not be used to treat the core symptoms of autism in children and adolescents.

**R 4.21:** mTOR inhibitors (everolimus, rapamycin) should not be used to treat the core symptoms of autism in children and adolescents.

**R 4.22:** Metformin should not be used to treat the core symptoms of autism in children and adolescents.

**R 4.23:** Cholinesterase inhibitors should not be used to treat the core symptoms of autism in children and adolescents.

**R 4.24:** Cannabinoids should not be used to treat the core symptoms of autism in children and adolescents.

**R 4.25:** Suramin should not be used to treat the core symptoms of autism in children and adolescents.

**R 4.26:** Naltrexone should not be used to treat the core symptoms of autism in children and adolescents.

**R 4.27:** Piracetam should not be used to treat the core symptoms of autism in children and adolescents.

5. ***Education***

**GPP 5.1:** Parents and caregivers should consult qualified professionals when considering educational interventions and school integration for their child with autism spectrum disorder, notably child psychiatrists and educational psychologists, as well as regional rehabilitation units that are informed about available educational accommodations.

**GPP 5.2:** Professionals should be guided by developmental and psycho-educational assessments, as well as by the provisions in place for preschool- and school-age children, when assisting and advising parents/caregivers on matters relating to education, schooling and various transitions.

**GPP 5.3:** When recommending an appropriate educational intervention and school placement, professionals should take into account, through the individualised education plan: (1) each child's developmental needs, preferences, strengths and particular interests, (2) the family context, and (3) the early intervention services and school measures available for children with particular developmental and/or educational needs.

**GPP 5.4:** Health and education professionals (educational psychologists) should ensure that parents/caregivers receive adequate support to make informed decisions that address the child's long-term educational needs, preferences and particular interests, taking into account their strengths and family environment.

**GPP 5.5:** Professionals should engage in information-sharing between institutions, where necessary, to ensure a shared understanding and to coordinate with parents/caregivers the support provided to the child.

**GPP 5.6:** Parents/caregivers should be informed about school integration measures as early as possible, generally from the time the child approaches 5 years of age and/or is enrolled in preschool (kindergarten).

**GPP 5.7:** At the point of transition to schooling, professionals should help parents obtain a comprehensive assessment of the needs of the child with autism spectrum disorder to ensure this transition is successful.

**GPP 5.8:** All schools in Tunisia are inclusive. Inclusive education involves creating learning environments that respond positively to the individual's different needs. The programme provides for a reduction in class sizes in inclusive classes, pending legislation to formalise this, as well as exceptional learning and assessment measures (reading instructions aloud, peer scribes, extra time, etc.) for these learners.

**GPP 5.9:** Teachers of students with autism spectrum disorder should have the knowledge and skills needed to provide accommodations and other educational support to these students in their classrooms. The level of information and mode of training should be tailored and personalised to their specific teaching and learning context.

**GPP 5.10:** Educational support accommodations in mainstream schools should be based on students' needs, not solely on their diagnoses.

**GPP 5.11:** Educational accommodations in the school setting offered to students with autism spectrum disorder should be determined by the education professionals working directly with the child (teachers, educational psychologist), in consultation with parents and, where necessary, health professionals (school physicians).

**GPP 5.12:** The school learning support assistant provides in-school support for children with specific needs who require personalised assistance. They contribute to implementing the individualised education plan. The learning support assistant should meet the specific support needs of the child with autism spectrum disorder in terms of school inclusion.

**GPP 5.13:** Referral of children with autism spectrum disorder who have particular educational needs and cannot be schooled in mainstream settings to specialised centres should be offered. There should be a sufficient number of such facilities, involving a multidisciplinary team of qualified teachers and allied health professionals. A specialised, individualised programme should be provided based on each child's needs and abilities.

**GPP 5.14:** Professionals should ensure that transition support is holistic, systematically planned and person-centred; this includes differentiated transition support based on the individual's identified needs.

**GPP 5.15:** Professionals should encourage and empower parents/caregivers to plan transitions in advance and to support their children in order to reduce any adverse consequences; this includes supporting the child, sharing information, and working closely with receiving schools.

**GPP 5.16:** Professionals should provide families with information about support groups, organisations and other sources of information recommended according to their needs.

**GPP 5.17:** During the transition from preschool to mainstream schooling, children with autism spectrum disorder should receive teaching and/or training in the following skills: communication, emotional and behavioural regulation, and social and adaptive skills.

**GPP 5.18:** Professionals working with students with autism spectrum disorder who are undergoing a transition should collaborate with schools and parents/caregivers to ensure these transitional stages go smoothly during the mainstream school pathway.

**GPP 5.19:** During the transition to post-school pathways, students with autism spectrum disorder and their parents/caregivers should receive information on post-school options and employment support.

6. ***Complementary and alternative treatments***

**GPP 6.1:** Professionals should be prepared to discuss the evidence on complementary and alternative medicine (CAM) with parents/caregivers of children and adolescents with autism spectrum disorder. Shared decision-making between professionals and parents/caregivers regarding CAM trials in autism is strongly encouraged, so that such trials are conducted with clear objectives and outcome measures. Parents/caregivers should not replace conventional interventions with CAM.

**R 6.2:** A gluten-free and casein-free diet is not recommended as a treatment for the core symptoms of autism in children and adolescents.

**GPP 6.3:** A healthy diet composed of fresh and varied foods is recommended for children and adolescents with autism spectrum disorder. Professionals should be informed of the recommended daily intake of vitamins, minerals and other supplements for children and adolescents according to age. They should be able to discuss with parents/caregivers the possible advantages and disadvantages of various supplements and their dosages.

**R 6.4:** The ketogenic diet is not recommended for treating the core symptoms of autism spectrum disorder in children and adolescents. However, in those with comorbid drug-resistant epilepsy, this diet may be considered. Initiation and monitoring of the ketogenic diet should be undertaken by a dietitian.

**R 6.5:** Camel milk is not recommended as a treatment for the core symptoms of autism in children and adolescents.

**R 6.6:** Vitamin supplementation (of any type) is not recommended for treating the core symptoms of autism in children and adolescents.

**GPP 6.7:** Children and adolescents with autism spectrum disorder who present with symptoms suggestive of a vitamin, mineral, amino acid or other nutritional deficiency should be assessed, treated and monitored in accordance with appropriate clinical guidelines.

**R 6.8:** Vitamin B6 is not recommended as a treatment for the core symptoms of autism in children and adolescents.

**R 6.9:** Folinic acid is not recommended for treating the core symptoms of autism in children and adolescents. Further research is needed to establish the potential benefits of this treatment.

**R 6.10:** Vitamin B12 is not recommended as a treatment for the core symptoms of autism in children and adolescents.

**R 6.11:** Supplementation with zinc, magnesium, iron or any other mineral is not recommended as a treatment for the core symptoms of autism in children and adolescents.

**R 6.12:** Amino acid supplementation is not recommended for treating the core symptoms of autism in children and adolescents.

**R 6.13:** Omega-3 fatty acids, in any presentation or combination (including with phosphatidylserine), are not recommended for treating the core symptoms of autism in children and adolescents. Further research is needed to establish the potential benefits of this treatment.

**R 6.14:** Probiotics are not recommended for treating the core symptoms of autism in children and adolescents. Further research is needed to demonstrate the potential benefits of probiotics in treating autism.

**R 6.15:** Secretin and digestive enzymes are not recommended as a treatment for the core symptoms of autism in children and adolescents.

**R 6.16:** Sulforaphane is not recommended for treating the core symptoms of autism in children and adolescents.

**R 6.17:** Coenzyme Q10 is not recommended for treating the core symptoms of autism in children and adolescents.

**R 6.18:** Antimicrobial therapy should not be used to treat the core symptoms of autism in children and adolescents, given the potential risk of harm and the absence of evidence of benefit.

**R 6.19:** Faecal microbiota transplant therapy should not be used to treat children and adolescents with autism spectrum disorder, given the risk of harm and the absence of evidence of benefit.

**R 6.20:** Helminth therapy (in any form) should not be used to treat children and adolescents with autism spectrum disorder, given the risk of harm and the absence of evidence of benefit.

**R 6.21:** Mesalazine is not recommended as a treatment for the core symptoms of autism in children and adolescents.

**R 6.22:** Immunoglobulin therapy (regardless of route of administration) should not be used to treat children and adolescents with autism spectrum disorder, given the risk of harm and the absence of evidence of benefit.

**R 6.23:** Stem cell therapy (intravenous or intrathecal) should not be used to treat children and adolescents with autism spectrum disorder, given the risk of harm.

**R 6.24:** Hyperbaric oxygen therapy should not be used to treat children and adolescents with autism spectrum disorder, given the risk of harm and the absence of evidence of benefit.

**R 6.25:** Chelation therapy should not be used to treat children and adolescents with autism spectrum disorder, given the risk of harm and the absence of evidence of benefit.

**R 6.26:** Neurofeedback is not recommended as a treatment for the core symptoms of autism in children and adolescents. Further research is needed to determine the potential benefits of this technique.

**R 6.27:** Vagus nerve stimulation should not be used to treat children and adolescents with autism spectrum disorder, as there is a risk of harm with no evidence of benefit.

**R 6.28:** Transcranial direct current stimulation is not recommended for treating the core symptoms of autism in children and adolescents.

**R 6.29:** Auditory integration therapy and other sound-based therapies are not recommended as a treatment for the core symptoms of autism in children and adolescents.

**R 6.30:** Music therapy may be recommended as a complementary approach in children and adolescents with autism spectrum disorder. There is a moderate level of evidence for improvements in overall functioning, quality of life and autism severity.

**R 6.31:** Dance/movement therapy is not recommended as a treatment for the core symptoms of autism in children and adolescents. Further studies are needed to establish any potential therapeutic benefits.

**GPP 6.32:** Dance and movement therapy may be offered to improve the health and well-being of these children.

**R 6.33:** Art therapy is not recommended as a treatment for the core symptoms of autism in children and adolescents. Further studies are needed to establish its potential benefits.

**GPP 6.34:** Art therapy may be offered to improve social engagement in these children.

**R 6.35:** Vision therapy is not recommended as a treatment for the core symptoms of autism spectrum disorder in children and adolescents. However, visuomotor exercises may be selectively offered to children with autism spectrum disorder who have visual difficulties, as such exercises have been shown to be effective in improving social communication and reducing repetitive behaviours in these children.

**R 6.36:** Aromatherapy should not be used to treat the core symptoms of autism spectrum disorder in children and adolescents, given the potential for harm.

**R 6.37:** Acupuncture is not recommended for treating the core symptoms of autism spectrum disorder in children and adolescents.

**R 6.38:** Massage is not recommended as a treatment for the core symptoms of autism spectrum disorder in children and adolescents.

**GPP 6.39:** Massage may be offered to improve sleep disturbances in these children.

**R 6.40:** Chiropractic, osteopathy and craniosacral therapy should not be used to treat children and adolescents with autism spectrum disorder, given the existing risk of harm.

**R 6.41:** Children and adolescents with autism spectrum disorder are recommended to engage in a variety of physical activities, at an intensity and frequency appropriate for their age, as indicated in the WHO guidelines on physical activity and sedentary behaviour.

**R 6.42:** Animal-assisted therapy is not recommended as a treatment for the core symptoms of autism in children and adolescents. Further research is needed to establish its potential benefits.

**GPP 6.43:** Animal-assisted therapy may be considered as part of overall care for certain children and adolescents with autism spectrum disorder, with the aim of improving their general well-being.

**R 6.44:** Mindfulness is not recommended as a treatment for the core symptoms of autism in children and adolescents. However, it may be considered as part of overall care for certain children and adolescents with autism spectrum disorder, with the aim of improving general well-being.

**R 6.45:** Facilitated communication should not be used to treat children and adolescents with autism spectrum disorder, as there is no evidence of benefit.

7. ***Autism and associated conditions***

**GPP 7.1:** Children and adolescents with autism spectrum disorder should be followed up at regular intervals and in a holistic manner (taking the whole person into account), as they are at increased risk of neuropsychological, adaptive and academic learning difficulties, in addition to certain physical conditions. Assessments and evaluations should be considered whenever abnormalities are identified in these areas.

**R 7.2:** Professionals should be aware that children and adolescents with autism spectrum disorder may present significant delays in adaptive skills, even in the absence of cognitive delay. Adaptive functioning should be assessed and monitored using standardised measures to address the child's functional needs.

**R 7.3:** Children and adolescents with autism spectrum disorder who present with academic difficulties should undergo assessments relevant to their learning needs. This will help guide parents and educators regarding further diagnostic investigations, specific interventions and support needed, as well as learning access provisions.

**R 7.4:** Professionals should be aware that the incidence of attention-deficit/hyperactivity disorder is high among children and adolescents with autism spectrum disorder. When symptoms of attention-deficit/hyperactivity disorder are present, particularly after age 5, prompt screening and referral for a thorough diagnostic assessment using validated measures should take place, to facilitate early management.

**R 7.5:** Professionals should be aware that children and adolescents with autism spectrum disorder are also prone to motor difficulties. Formal screening and diagnosis of developmental coordination disorder (DCD), using standardised tests, should be undertaken in individuals showing slowness and clumsiness in the acquisition and execution of motor coordination skills beyond the preschool period.

**R 7.6:** Professionals should be aware of the need to assess language, learning, and other associated developmental disorders in children and adolescents on the autism spectrum in order to provide appropriate support. Parents/caregivers should receive guidance regarding these associated disorders and their potential impact on the child's learning and adaptive behaviour.

**R 7.7:** Children with autism who present with global developmental delay should be reassessed toward the end of the preschool period to check for the presence of intellectual disability. A diagnosis of global developmental delay should not be retained once the child is older than 5 years.

**R 7.8:** Children and adolescents with autism spectrum disorder who present with sensory processing difficulties should undergo a holistic assessment (questionnaires, direct ecological observations), carried out by a qualified professional using validated tools.

**R 7.9:** Children and adolescents with autism spectrum disorder who present with symptoms related to mental health conditions (e.g., depression, anxiety) that affect their daily functioning should undergo a more thorough assessment. Professionals should be able to detect mental health conditions associated with autism and should receive adequate training on this subject.

**GPP 7.10:** Professionals should be alert to a potential association between gender variance and autism. Children and adolescents with autism who present with gender variance concerns may need referral for further assessment and support tailored to their socio-emotional needs.

**R 7.11:** Health professionals should be aware of the increased prevalence of eating disorders among children and adolescents with autism spectrum disorder. These disorders may be linked to various factors, including feeding dysfunction, sensory sensitivity, adaptive delays, behavioural problems and cognitive difficulties. They may also include pica (ingestion of non-food items), rumination (regurgitating and re-chewing previously swallowed food), obesity and food neophobia (fear of new foods). These disorders should be assessed when necessary.

**GPP 7.12:** Children with autism spectrum disorder who have a history of early-onset, persistent regulatory problems (feeding difficulties, sleep problems and excessive crying persisting beyond 3 months of age) may be at higher risk of eating disorders and should be monitored for these conditions.

**R 7.13:** Sleep disorders are common in children and adolescents with autism spectrum disorder. Health professionals should monitor sleep patterns and treat sleep disorders, as poor sleep quality affects the well-being of the individual and their caregivers and is associated with various adverse consequences, including an increased risk of overweight and obesity.

**GPP 7.14:** Health professionals should be aware of the possibility of earlier pubertal onset and a higher risk of precocious puberty in girls with autism spectrum disorder compared with neurotypical girls. Routine monitoring and referral for further investigation should be arranged in the presence of any concerning sign or symptom.

**GPP 7.15:** There is an increased risk of obesity in children and adolescents on the autism spectrum, as differences in social interaction, motor coordination challenges and psychosocial issues related to autism can contribute to increased sedentary behaviour. Professionals should encourage preventive measures to reduce these risks and should monitor weight, height, body mass index and any significant changes in growth percentiles over time.

**R 7.16:** Health professionals should be aware that children and adolescents with autism spectrum disorder have a higher rate of gastrointestinal disorders. Those with persistent, chronic or recurrent symptoms such as recurrent colic or abdominal pain, vomiting, non-specific diarrhoea or constipation should be referred for a thorough assessment.

**GPP 7.17:** Health professionals should be aware that children and adolescents with autism spectrum disorder who have gastrointestinal disorders may present with atypical behavioural disturbances indicative of an acute abdominal condition. Investigation for a gastrointestinal disorder should be considered for children and adolescents with autism spectrum disorder who show unexplained, persistent or sudden atypical behavioural symptoms.

**GPP 7.18:** Health professionals should be alert to potential nutritional problems in children and adolescents with autism spectrum disorder. They should monitor growth and nutritional status, given the increased risk of metabolic and psychosocial complications linked to overweight or underweight. In cases of inadequate growth or obesity, the child should be referred to a specialist.

**GPP 7.19:** Health professionals should be aware of the possible co-occurrence of epilepsy in children and adolescents with autism spectrum disorder, and should refer the patient to a paediatric neurologist in the presence of genuine clinical seizures, features suggestive of seizures, and/or cognitive regression.

**R 7.20:** A comprehensive hearing assessment is recommended for all children with suspected autism, so as not to delay the diagnosis of an associated hearing impairment, and to allow for appropriate management in cases of comorbidity between autism and hearing impairment.

**GPP 7.21:** There is a high incidence of visual disorders (e.g., strabismus, refractive errors, anisometropia and amblyopia) among children and adolescents with autism spectrum disorder, which may present as unexplained behavioural disturbances or academic difficulties. These children should receive routine vision screening and monitoring for visual disorders, and should be referred to a specialist when needed.

**GPP 7.22:** Professionals should be aware that the incidence of cavities and gum problems is higher among children and adolescents with autism spectrum disorder, and that these problems are associated with food selectivity and eating disorders. Routine dental screening and care should be offered and facilitated, particularly in the presence of untreated or insufficiently treated cavities and gum problems.

8. ***Follow-up and prognosis***

**GPP 8.1:** Interventions aimed at promoting positive parenting, both maternal and paternal, should be encouraged. This should include support structures that enable parents to understand their child's/adolescent's autism and its associated challenges as early as possible. Parental participation in training on managing children's challenging behaviours, as well as in support groups, should be encouraged.

**GPP 8.2:** Interventions for children and adolescents with autism spectrum disorder should address these individuals' needs across their lifespan. This includes adaptive functioning, emotional well-being and academic achievement, in order to improve long-term quality of life. The goals of the child/adolescent and their family should also be taken into account.

**GPP 8.3:** Systematic planning should be put in place to manage major transitions for children and adolescents with autism spectrum disorder. This planning should be proactive, holistic and person-centred. It is particularly important to plan early transitions for adolescents with autism spectrum disorder before they leave mainstream school or specialised centres.

9. ***Support for caregivers and families***

**GPP 9.1:** Professionals should be able to recognise the impact of autism spectrum disorder on parents and families in terms of social, economic, physical and mental well-being, in order to provide optimal support.

**GPP 9.2:** Professionals should provide caregivers with information tailored to the age and developmental needs of the child or adolescent with autism spectrum disorder. They should also help them access appropriate services.

**GPP 9.3:** Professionals should adopt a collaborative, family-centred approach to help parents/caregivers achieve optimal therapeutic outcomes.

**GPP 9.4:** Professionals should assess the emotional well-being of parents/caregivers, as well as their coping capacity, in order to provide the support needed for holistic care.

**GPP 9.5:** Professionals should support parents/caregivers at every stage of the child's and adolescent's life, particularly for future care planning.

**R 9.6:** Training and education for parents/caregivers should be integrated into intervention programmes for children and adolescents with autism spectrum disorder whenever possible, given the positive effects for both the child or adolescent and the caregiver.

10. ***Professional training***

**GPP 10.1:** Staff who interact with, or care for (directly or indirectly), children and adolescents with autism spectrum disorder should have access to information about autism. The scope and level of information should be tailored to the specific needs of professionals.
