# Supplementary material for: Development and contextual adaptation of the first national clinical practice guideline for autism spectrum disorder in children and adolescents in Tunisia
Source: Front Psychiatry. 2026 Oct 20;17:1926851. doi: 10.3389/fpsyt.2026.1926851 (PMC13727530; doi:10.3389/fpsyt.2026.1926851)
Supplement: Supplementary file 5 [file Table5.docx]

**Title: Development and contextual adaptation of the first national clinical practice guideline for autism spectrum disorder in children and adolescents in Tunisia**

Randaline Ayoub^1^**^,2*^**, Hela Ben Abid^1,2^, Soumaya Bourgou^3,4^, Imen Hadj Kacem^5^,  Soumeyya Halayem^6^, Khaoula Khemekhem^5^, Fatma Charfi^3,4^, Asma Bouden^6^

**Randaline Ayoub, MD (Corresponding author)**

Assistant Professor in Child and Adolescent Psychiatry, Fattouma Bourguiba University Hospital, Faculty of Medicine, University of Monastir, Monastir, Tunisia

Permanent Full poster address: Faculty of Medicine, University of Monastir, Avicenne Avenue, Monastir 5000, Tunisia

Phone: +216 21 481 979; Fax: +216 73 460 309

E-mail address: [ayoub.randaline@gmail.com](mailto:ayoub.randaline@gmail.com)

**Supplementary File S5: Tunisian ASD Clinical Practice Guideline Working Group**

**Steering Committee**

**President**
Bouden Asma – Child and Adolescent Psychiatry, Faculty of Medicine of Tunis, Razi Hospital, Tunis, Tunisia

**Vice-President**
Charfi Fatma – Child and Adolescent Psychiatry, Faculty of Medicine of Tunis, Mongi Slim Hospital, Tunis, Tunisia

**Methodological Team**

Ayoub Randaline – Child and Adolescent Psychiatry, Faculty of Medicine of Monastir, Fattouma Bourguiba Hospital, Monastir, Tunisia

Khemekhem Khaoula – Child and Adolescent Psychiatry, Faculty of Medicine of Sfax, Hédi Chaker Hospital, Sfax, Tunisia

**Writing Committee**

- **Group Leads**

Ben Abid Hela – Child and Adolescent Psychiatry, Faculty of Medicine of Monastir, Fattouma Bourguiba Hospital, Monastir, Tunisia

Bourgou Soumaya – Child and Adolescent Psychiatry, Faculty of Medicine of Tunis, Mongi Slim Hospital, Tunis, Tunisia

Halayem Soumeya – Child and Adolescent Psychiatry, Faculty of Medicine of Tunis, Razi Hospital, Tunis, Tunisia

Hadjkacem Imen – Child and Adolescent Psychiatry, Faculty of Medicine of Sfax, Hédi Chaker Hospital, Sfax, Tunisia

- **General Secretaries**

Ben Ltaifa Asma – Child and Adolescent Psychiatry, Faculty of Medicine of Tunis, Military Hospital of Tunis, Tunisia

Chaabane Imen – Child and Adolescent Psychiatry, National School and University Health Center, Tunis, Tunisia

Ferchichi Siwar – Clinical Psychology, Razi Hospital, Tunis, Tunisia

- **Working Group Members**

Abid Mohamed – Psychomotricity, Private Practice, Tunis, Tunisia

Achour Ahlem – Medical Genetics, Faculty of Medicine of Tunis, Charles Nicolle Hospital, Tunis, Tunisia

Ajmi Maha – Child and Adolescent Psychiatry, Faculty of Medicine of Monastir, Fattouma Bourguiba Hospital, Monastir, Tunisia

Askri Wael – Child and Adolescent Psychiatry, Faculty of Medicine of Tunis, Razi Hospital, Tunis, Tunisia

Bagbag Khadija – Child and Adolescent Psychiatry, Private Practice, Tunis, Tunisia

Ben Chouchene Nourhen – Speech and Language Therapy, Leader Rehabilitation Center, Tunisia

Ben Elbey Amina – Child and Adolescent Psychiatry, Regional Rehabilitation Unit, Sfax, Tunisia

Ben Hamouda Abir – Child and Adolescent Psychiatry, Faculty of Medicine of Tunis, Mongi Slim Hospital, Tunis, Tunisia

Ben Touhami Donia – Child and Adolescent Psychiatry, Faculty of Medicine of Sfax, Hédi Chaker Hospital, Sfax, Tunisia

Ben Yahia Ayadi Houda – Clinical Psychology and Neuropsychology, Razi Hospital, Tunis, Tunisia

Boudabous Jaweher – Child and Adolescent Psychiatry, Faculty of Medicine of Sfax, Hédi Chaker Hospital, Sfax, Tunisia

Chekili Wafa – Health Sciences, Higher School of Health Sciences and Techniques, Tunis, Tunisia

Cherif Emna – Child and Adolescent Psychiatry, Faculty of Medicine of Tunis, Razi Hospital, Tunis, Tunisia

Chtioui Emna – Child and Adolescent Psychiatry, Private Practice, Tunis, Tunisia

Daoud Mona – Child and Adolescent Psychiatry, Faculty of Medicine of Tunis, Mongi Slim Hospital, Tunis, Tunisia

Hajri Melek – Child and Adolescent Psychiatry, Faculty of Medicine of Tunis, Razi Hospital, Tunis, Tunisia

Hakiri Abir – Psychiatry, Faculty of Medicine of Tunis, Razi Hospital, Tunis, Tunisia

Hanachi Rimeh – Clinical Psychology and Psychotherapy, Private Practice, Tunisia

Hsairi Guidara Ines – Neurology, Regional School and University Health Center, Sfax, Tunisia

Jelili Selima – Child and Adolescent Psychiatry, Private Practice, Tunis, Tunisia

Kaffel Nadia – Otorhinolaryngology, Faculty of Medicine of Tunis, Mongi Slim Hospital, Tunis, Tunisia

Kammoun Bouaicha Hanene – Child and Adolescent Psychiatry, Regional Hospital of Sidi Bouzid, Tunisia

Kammoun Wiem – Child and Adolescent Psychiatry, Private Practice, Tunis, Tunisia

Karoui Sana – Medical Genetics, Faculty of Medicine of Tunis, Mongi Slim Hospital, Tunis, Tunisia

Kebaili Krid Rim – Child and Adolescent Psychiatry, Regional School Health Center, Sfax, Tunisia

Lamloum Hadrich Sawssen – Speech and Language Therapy, Higher School of Health Sciences, Tunis, Tunisia

Louati Nesrine – Occupational Therapy, Leader Rehabilitation Center, Tunisia

Makni Ayadi Fatma – Biochemistry, Faculty of Medicine of Sfax, Habib Bourguiba Hospital, Sfax, Tunisia

Marrakchi Jihen – Otorhinolaryngology, Faculty of Medicine of Tunis, Menzel Bourguiba Hospital, Tunisia

Miladi Zouhour – Pediatric Neurology, National Institute of Neurology Mongi Ben Hmida, Tunis, Tunisia

Moalla Yosr – Child and Adolescent Psychiatry, Faculty of Medicine of Sfax, Hédi Chaker Hospital, Sfax, Tunisia

Nabli Ahmed – Occupational Therapy and Digital Health, ReMotion, Lille, France

Nouira Ons – Child and Adolescent Psychiatry, Private Practice, Tunis, Tunisia

Snoussi Emna – Psychomotricity and Clinical Psychology, Private Practice, Tunis, Tunisia

Souid Ons – Child and Adolescent Psychiatry, Private Practice, Monastir, Tunisia

Souyah Najla – Pediatrics, Faculty of Medicine of Sousse, Farhat Hached Hospital, Sousse, Tunisia

Tej Amel – Pediatrics, Faculty of Medicine of Sousse, Farhat Hached Hospital, Sousse, Tunisia

Zaraa Ahmed Adam – Clinical Psychology and Psychotherapy, Tunisia

**External Review Group**

Abbes Zeineb – Child and Adolescent Psychiatry, Faculty of Medicine of Tunis, Razi Hospital, Tunis, Tunisia

Amara Mejda – Public Health, Ministry of Health, Tunisia

Aouididi Dorra – Pharmacy, Razi Hospital, Tunis, Tunisia

Ben Jemaa Lamia – Medical Genetics, Faculty of Medicine of Tunis, Mongi Slim Hospital, Tunis, Tunisia

Ben Khalfa Adnene – Primary Care, Sidi Hassine Health Center, Tunisia

Benrhouma Hanene – Pediatric Neurology, National Institute of Neurology, Tunis, Tunisia

Chebbi Hichem – Education Expert, Ministry of Education, Tunisia

Guedria Asma – Child and Adolescent Psychiatry, Faculty of Medicine of Monastir, Monastir, Tunisia

Hmida Hentati Nedia – Neonatology, Faculty of Medicine of Sfax, Sfax, Tunisia

Jazi Saoussen – Rehabilitation Medicine, Nabeul, Tunisia

Laaribi Hela – Child and Adolescent Psychiatry, Private Practice, Sfax, Tunisia

Naouar Hanene – School Psychology, Manouba, Tunisia

Meddouri Samiha – Child and Adolescent Psychiatry, Faculty of Medicine of Tunis, Tunis, Tunisia

Rafrafi Rym – Psychiatry, Faculty of Medicine of Tunis, Mongi Slim Hospital, Tunis, Tunisia

Triki Charfi Chahnez – Pediatric Neurology, Faculty of Medicine of Sfax, Sfax, Tunisia

F’s Family: Individual with Autism Spectrum Disorder and caregivers

**Guideline Endorsement Committee (INEAS)**

Adel Khelil – INEAS, Tunisia

Hella Ouertatani – INEAS, Tunisia

Mohamed Ben Hamouda – INEAS, Tunisia

Hèla Ayadi – Child and Adolescent Psychiatry, Sfax, Tunisia

Ahlem Gzara – Public Health, Tunisia

Hela Ouennich – Child and Adolescent Psychiatry, Tunisia
